# Macrophage Membrane-Engineered Biomimetic Nanoplatform Enables Immune Evasion and Immunomodulation for Enhanced Chemo-Photodynamic Therapy

**DOI:** 10.1021/acs.biomac.6c01045

**Published:** 2026-06-26

**Authors:** Tugba Gencoglu-Katmerlikaya, Aydan Dag

**Affiliations:** † Department of Biotechnology, Institute of Health Sciences, 221265 Bezmialem Vakif University, 34093 Istanbul, Turkey; ‡ Department of Pharmaceutical Chemistry, Faculty of Pharmacy, Bezmialem Vakif University, 34093 Istanbul, Turkey; § Pharmaceutical Application and Research Center, Bezmialem Vakif University, 34093 Istanbul, Turkey

## Abstract

Triple-negative breast cancer (TNBC) remains one of the most aggressive breast cancer subtypes with insufficient therapeutic opportunities and poor clinical outcomes. This challenge can be addressed to a great extent by immunotherapy, but its clinical efficacy is substantially limited by the immunosuppressive tumor microenvironment. To overcome these barriers, an intelligent M1-type macrophage membrane camouflaged biomimetic nanoplatform based on cholesterol-functionalized glycopolymers bearing a photodynamic therapy (PDT)-active unit protoporphyrin IX (PpIX) and pH-responsive linkages for doxorubicin (Dox) conjugation has been developed to suppress TNBC through the combination of chemo-photodynamic therapy and immune remodeling. The therapeutic efficacy of the NP-Dox@M1 nanoplatform against metastatic TNBC was systematically evaluated in vitro through a comparative analysis of membrane-coated (NP@M1) versus uncoated (NP) constructs and drug-loaded (NP-Dox) versus drug-free formulations under conditions with or without light irradiation. The NP-Dox@M1 nanoplatform enables the synergistic combination of enhanced chemo-photodynamic therapy and immune regulation by promoting the polarization of macrophages toward an M1-like phenotype and reducing macrophage uptake, demonstrating potential immune evasion for the effective treatment of TNBC.

## Graphical abstract

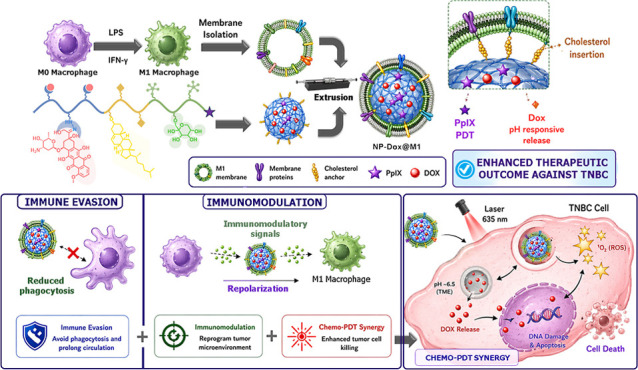

## Introduction

Breast cancer is the most frequently diagnosed malignancy and remains a leading cause of cancer-associated mortality among women worldwide. According to recent statistics reported in the American Cancer Society journal *CA: A Cancer Journal for Clinicians*, it ranked first in incidence and fourth in mortality in 2025.^1^ Among its subtypes, triple-negative breast cancer (TNBC) is one of the most aggressive forms and is associated with the poorest prognosis.^2–4^ Although surgery, radiotherapy, chemotherapy, and immunotherapy are currently employed in its treatment, these approaches remain insufficient and fail to achieve satisfactory clinical outcomes.^5^ The inadequacy of conventional cancer treatment methods due to their lack of targeting, their off-target toxicity, and their potential impact on healthy cells as well as cancer cells has led to a growing number of studies on novel, targeted, and biomimetic nanoplatforms integrating immunomodulation with combined treatment strategies.^6–9^

In this regard, membrane engineering for the synthesis of biomimetic nanoplatforms is an important field of study that allows cell-derived systems to acquire new functionalities by manipulating the physical and chemical properties of their membranes.^10,11^ This strategy provides a powerful foundation for the design of advanced biomimetic nanoplatforms with an enhanced targeting capability, immune interaction, and therapeutic efficacy.

Both genetic and nongenetic engineering approaches can be employed to fabricate cell-derived biomimetic nanoplatforms by modulating and reprogramming the membrane surface architecture to stand out as an innovative approach shaping the future of health sciences in various biological applications.^12^ While genetic engineering allows for the permanent reprogramming of cells, this process is time-consuming and complex in terms of regulation, so researchers have turned to nongenetic alternatives. Among nongenetic strategies, hydrophobic insertion, which is based on the spontaneous embedding of amphiphilic molecules into the lipid bilayer, induces functional modifications on cell membranes.^13^ With this approach, biomolecules such as antibodies, peptides, or therapeutic agents can be directly integrated into the cell membrane using hydrophobic anchor molecules such as cholesterol, phospholipids, or long-chain alkanes, thereby enhancing target-specific binding and immunological functionality. Therefore, hydrophobic insertion stands out as a complementary method to genetic approaches in cell engineering in terms of practical feasibility, flexibility, and clinical adaptability.

Different applications of membrane engineering offer innovative approaches to both diagnostic and therapeutic processes through cell membrane redesign. For example, altering the biophysical properties of tumor cell membranes facilitates the selective uptake and efficient delivery of drugs to intracellular targets.^14^ Nanotechnology-based membrane coatings contribute to the protection of healthy tissues by enabling the targeted delivery of chemotherapeutic agents.^15^

Furthermore, engineered cell membranes derived from immune cells such as macrophages, dendritic cells, or lymphocytes can be utilized in immunotherapies to enhance immune recognition of tumor cells and strengthen antitumor immune responses.^16^ Therefore, membrane engineering, especially of immune cells, holds significant strategic potential for drug delivery systems, immunotherapy, and early diagnostic applications in cancer treatment. For drug delivery applications, engineered immune cell membranes can be utilized to fabricate membrane-coated nanoplatforms such as polymeric nanoparticles, inorganic nanoparticles, liposomes, metal–organic frameworks (MOFs), and micelles, which collectively mimic the intrinsic biological properties of immune cells.^17–19^

Macrophage membrane-coated nanoplatforms have been explored as a biomimetic strategy to improve tumor targeting by leveraging the inherent inflammation-homing and immune-evasive properties of macrophages, enabling enhanced accumulation of therapeutic agents at tumor and metastatic sites.^20,21^ In breast cancer models, such systems have been shown to increase delivery efficiency to metastatic lesions and improve overall therapeutic outcomes by mimicking natural macrophage behavior.^21^ Zhang et al. fabricated nanoparticles cloaked with membranes derived from macrophages to combine the innate homing ability of macrophages with nanoparticle-based chemotherapy. Additionally, macrophage–cancer hybrid membrane-coated nanoparticles have been reported to enhance accumulation in metastatic lesions and significantly inhibit lung metastasis in breast cancer models, highlighting the potential of biomimetic membrane engineering for metastatic cancer therapy.^22^

The differentiation of macrophages into their active forms by stimulating them with LPS and IFNγ, as well as the subsequent use of membranes derived from these activated macrophages for coating nanoplatforms, has also been evaluated by researchers as an advanced targeted approach.^23^ Recent studies have highlighted the potential of M1-type macrophage membranes for coating of nanoplatforms as biomimetic drug delivery systems due to their immune evasive properties and pro-inflammatory characteristics. In a study, nanoplatforms cloaked with M1 macrophage membranes were reported to achieve enhanced penetration into tumor organoids and improved anticancer efficacy, demonstrating the therapeutic promise of M1 membrane engineering in cancer treatment.^24^ Similarly, macrophage membrane-coated nanoplatforms have been developed to remodel the tumor microenvironment by reprogramming tumor-associated macrophages toward a pro-inflammatory M1 phenotype and enhancing T cell-mediated antitumor immunity, thereby improving the efficacy of combinational therapy.^25^

The development of synergistic and combinational cancer therapies has attracted considerable attention as an effective strategy to overcome the limitations of conventional monotherapies, particularly through the integration of multifunctional nanoplatforms incorporating cell membrane coatings that enable immunomodulation, prolonged circulation, and enhanced tumor targeting.^26^ Biomimetic nanoplatforms coated with M1 macrophage membranes have been developed to enable tumor-targeted delivery, imaging-guided photothermal therapy, photodynamic therapy (PDT), and synergistic chemo-immunotherapy by improving both immune evasion and tumor accumulation.^27^ Membrane-coated nanoplatforms designed for controlled drug release in response to different stimuli, which improve tumor targeting beyond ligand-based strategies by exploiting tumor cell membrane properties, offer important advantages for cancer therapy.^28,29^

Glucose transporter (GLUT)-mediated targeting has gained increasing attention as an effective approach to exploit the enhanced glycolytic activity of cancer cells, thereby improving the specificity and uptake efficiency of nanotherapeutics within the tumor microenvironment.^30^ Building upon these advances, cell-membrane-derived nanocarriers present a unique opportunity to integrate GLUT targeting with immune modulation. Among these, fructose transporter 5 (GLUT5) has emerged as a promising target due to its upregulated expression in multiple cancer types, including breast cancer, and its role in supporting tumor growth and survival.^31–34^ This combinatorial design enables the simultaneous amplification of phagocytic cues and GLUT targeting within the tumor microenvironment.

Here, we report a modular, M1 macrophage cell membrane-engineered biomimetic nanoplatform (NP-Dox@M1) assisted by cholesterol insertion that integrates GLUT targeting with dual therapeutic strategies combining chemo-photodynamic therapy. Our findings indicate that NP-Dox@M1 markedly promotes macrophage cell-membrane-driven cellular internalization activity while simultaneously strengthening antitumor responses, underscoring their robust therapeutic efficacy in triple-negative breast cancer. Distinct from conventional approaches that depend on extensive chemical functionalization, this strategy leverages membrane-derived bioactivity, thereby avoiding complex synthetic procedures and ensuring the preservation of protein functionality. In addition, the modular nature of this platform enables the rational integration of diverse engineered membranes, providing a versatile framework for tailoring dual-signal immunomodulatory and immune evasive properties in a disease-specific manner.

## Experimental Section

### Polymer Synthesis

#### Synthesis of the P­(iFMA) Homopolymer (P1)

The P­(*i*FMA) homopolymer was synthesized using the isopropylidene-protected fructose methacrylate (*i*FMA) monomer with a [Monomer]/[RAFT]/[Initiator] ratio of [40]:[1]:[0.1]. Briefly, *i*FMA (0.8 g, 2.43 mmol) and the alkyne functional reversible addition–fragmentation chain transfer (RAFT) agent (26.0 mg, 0.81 mmol) were put into a vial and dissolved with 3 mL of toluene. Azobis­(isobutyronitrile) (AIBN) (1.7 mg, 10.15 μmol) was added from the stock solution, and the vial was quickly sealed with a rubber septum. The reaction mixture was degassed with nitrogen flow for 50 min at 0 °C. Then, the vial was placed in an oil bath set at 70 °C, and polymerization was carried out for 16 h. The polymer was purified by precipitating into hexane twice to remove the unreacted monomer.^35^ The chemical structure and molecular weight characteristics of the synthesized polymer were confirmed by proton nuclear magnetic resonance (^1^H NMR) spectroscopy, Fourier Transform Infrared (FT-IR) spectroscopy, and Gel Permeation Chromatography (GPC).

#### Synthesis of the P­(iFMA-*b*-(CMA-*co*-bHMA)) Polymer (P2)

P­(iFMA) P1 was used as a macroRAFT agent, and a second block consisting of a copolymer of cholesterol methacrylate (CMA, M1) and *tert*-butyloxycarbonyl-protected hydrazine methacrylate (bHMA, M2) was synthesized via RAFT polymerization. The feed ratio of [M1:M2]/[macroRAFT]/[initiator] was set as [15:8]:[1]:[0.125]. MacroRAFT (0.25 g, 31.8 μmol), CMA (0.22 g, 0.48 mmol), and bHMA (50.0 mg, 0.26 mmol) were weighed and added to a vial and then dissolved in a mixture of toluene (2 mL) and 2,2,2-trifluoroethanol (TFE, 0.1 mL). AIBN (0.65 mg, 3.9 μmol) was added from the stock solution, and the vial was quickly sealed with a rubber septum. The reaction mixture was degassed with nitrogen flow for 50 min at 0 °C. Then, the vial was placed in an oil bath set at 70 °C, and polymerization was carried out for 16 h. The polymer was purified by precipitating into hexane twice to remove the unreacted monomer.^36,37^ The chemical structure and molecular weight characteristics of P2 were confirmed by ^1^H NMR, FT-IR spectroscopy, and GPC.

#### Synthesis of P­(iFMA-*b*-(CMA-*co*-bHMA))-PpIX (P3)

The alkyne-terminated polymer (0.2 mg) was dissolved in 2 mL of a 1:1 mixture of anhydrous dimethylformamide (DMF) and tetrahydrofuran (THF). Protoporphyrin IX (PpIX) (2.0 eq relative to the alkyne group) was then added to the solution. Separately, *N,N,N*′*,N*″,*N*″-pentamethyldiethylenetriamine (PMDETA) (10 equiv) and CuBr (2 equiv) were then added to the reaction mixture. Three freeze–pump–thaw cycles were performed to degas the system and remove dissolved oxygen. The reaction mixture was then stirred at room temperature for 24 h.

After completion of the reaction, the crude mixture was passed through a short column packed with neutral alumina to remove the copper catalyst and residual impurities. The collected solution was then concentrated under reduced pressure to a smaller volume. The polymer conjugate was then made to precipitate by adding the solution slowly to excess cold diethyl ether, all while stirring vigorously. The resulting precipitate was collected by filtration, washed with extra diethyl ether to remove any leftover solvent and unreacted small molecules, and then dried under vacuum to produce the purified polymer as a solid.^38^ The P3 polymer was characterized by ^1^H NMR, FT-IR spectroscopy, and GPC.

#### Synthesis of P­(FMA-*b*-(CMA-*co*-HMA))-PpIX (P4)

The isopropylidene (*i*) and *tert*-butoxy carbonyl (*b*) protecting groups-bearing P­(*i*FMA-*b*-(CMA-*co*-*b*HMA))-PpIX polymer (114 mg) was dissolved in a chloroform/water mixture (9:1, v/v). Trifluoroacetic acid (TFA) (4.3 mL) was then added dropwise to the solution while being stirred at room temperature. The reaction mixture was stirred for 16 h to allow the acid-catalyzed hydrolysis of the *i* and *b* protecting groups. Once the reaction had finished, the solvent was then removed under reduced pressure, and the resulting polymer was precipitated into cold diethyl ether to produce the P­(FMA-*b*-(CMA-*co*-HMA))-PpIX (P4) deprotected polymer.^35^ The chemical structure and molecular weight characteristics of P4 were confirmed by ^1^H NMR, FT-IR spectroscopy, and GPC.

#### Synthesis of P­(iFMA-*b*-(CMA-*co*-HMA-Dox))-PpIX (P5)

Doxorubicin hydrochloride, Dox (23 mg, 39.4 μmol), was dissolved in 0.5 mL of dimethyl sulfoxide (DMSO) and neutralized by adding triethylamine (1.2 equiv) to liberate the free amine group. In a separate flask, the P4 polymer (50 mg) was dissolved in 1 mL of DMSO. The Dox solution was then added dropwise to the polymer solution while stirring continuously. The reaction mixture was stirred at room temperature for 24 h under a nitrogen atmosphere to allow the conjugation of Dox on the polymer backbone. The reaction mixture was transferred to a dialysis membrane (MWCO 3.5 kDa) and dialyzed against distilled water for 48 h to remove the unreacted Dox. The water was replaced every 6 h. The purified conjugate was then lyophilized to obtain P­(*i*FMA-*b*-(CMA-*co*-HMA-Dox))-PpIX (P5).^37^ The P5 polymer was characterized by ^1^H NMR, FT-IR spectroscopy, and GPC.

### Membrane Isolation

RAW264.7 cells were differentiated into the M1 phenotype by treatment with 0.5 μg of lipopolysaccharide (LPS) and interferon-gamma (IFNγ) for 24 h prior to membrane isolation. Macrophages differentiated into the M1 phenotype were stained with FITC anti-mouse CD80 and PE anti-mouse CD86 antibodies and analyzed by flow cytometry to confirm their differentiation to the M1-type.^39^ The cell membrane of RAW264.7 macrophage cells was isolated using a membrane protein extraction kit (Mem-PER Plus, Thermo Scientific) by using the published procedure. For immunoblotting, total proteins from RAW264.7 cells were extracted using the RIPA buffer. Protein concentration was determined using a bicinchoninic acid (BCA) assay kit, and the isolated proteins were analyzed by Western blotting. Briefly, proteins were denatured at 95 °C, and 25 μg of protein per well was loaded onto a 10% sodium dodecyl sulfate polyacrylamide gel electrophoresis (SDS-PAGE) gel. After electrophoresis, proteins were transferred onto a poly­(vinylidene fluoride) (PVDF) membrane using a semidry transfer system. The membranes were then blocked with 5% nonfat dry milk in the tris-buffered saline with Tween 20 (TBST) for 1 h and incubated overnight at 4 °C with primary antibodies (horseradish peroxidase (HRP)-conjugated GAPDH 1:1000, abcam; Na + K+ ATPase 1:2000, abclonal; transferrin receptor 1 (TFR1) 1:1000, abclonal). After washing, membranes were incubated for 1 h with an antirabbit secondary antibody (1:5000 dilution). Protein bands were monitored using a chemiluminescent substrate and analyzed with ImageJ software.^40^

### Preparation of Biomimetic Nanoplatforms

The nanoprecipitation technique was used to prepare both Dox-free nanoplatforms (NP and NP@M1) and Dox-loaded nanoplatforms (NP-Dox and NP-Dox@M1) by employing P4 and P5 polymers, respectively. In brief, 5.0 mg of the P4 or P5 polymer was dissolved in 0.5 mL of dimethyl sulfoxide (DMSO) and stirred at room temperature for 30 min. The resulting polymer solution was then gradually added into 4.5 mL of ultrapure water by using a syringe pump to form NP or NP-Dox. These mixtures were stirred at room temperature for 24 h, followed by dialysis (MWCO 3.5 kDa) against ultrapure water to eliminate DMSO.^38^

M1 membrane-coated nanoplatforms were subsequently prepared via an extrusion-based coating approach using NP and NP-Dox, yielding NP@M1 and NP-Dox@M1, respectively. Specifically, NP and NP-Dox were mixed with membrane proteins at a 1:1 (w/w) ratio and subjected to ultrasonic bath sonication for 5 min. The mixture was then passed through an Avanti mini-extruder. To maintain consistent concentrations, appropriate amounts of ultrapure water were added to NP and NP-Dox formulations. To quantify PpIX and Dox loading within the nanoplatforms, calibration curves were generated using UV–vis spectroscopy. The hydrodynamic size distribution and surface charge of the prepared nanoparticles were measured by dynamic light scattering (DLS), while their morphology and size were further characterized using transmission electron microscopy (TEM), and protein profiles of biomimetic nanoplatforms were analyzed by SDS-PAGE.^40^

### Stability of the Biomimetic Nanoplatform

The colloidal stability of NP-Dox@M1 was studied in PBS (pH 7.4) and complete cell culture medium at room temperature for 7 days. The particle size and ζ-potential were measured daily via DLS in order to monitor time-dependent variations in hydrodynamic diameter and surface charge.^41^

### Determination of the In Vitro Release Profile

The drug release profiles of NP-Dox and NP-Dox@M1 were evaluated in buffer media at pH 7.4 and pH 5.5. In brief, 1 mL of each formulation was loaded into a dialysis bag (MWCO 3.5 kDa), which was subsequently immersed in 20 mL of buffer solution and incubated at 37 °C. At defined time points, 2 mL of the external release medium was collected and replaced with an equal volume of fresh buffer. The concentration of released Dox was then quantified using UV–vis spectrometry.^31^

### Singlet Oxygen Generation

1,3-Diphenylisobenzofuran (DPBF) was employed as a probe to evaluate singlet oxygen (^1^O_2_) generation upon irradiation. NP-Dox@M1 and NP@M1 were prepared in DMSO at identical PpIX concentrations. The production of singlet oxygen was assessed by tracking the reduction in absorbance of DPBF at 414 nm as DPBF acts as a singlet oxygen scavenger. Initially, a DPBF solution (150 μM) was added to samples containing a fixed concentration of the photosensitizer (3.4 μM). For all formulations, the solvent composition was maintained at a DMSO/water ratio of 1:5 (v/v). Before light exposure, the baseline absorbance at 414 nm was recorded using UV–vis spectroscopy. Subsequently, the samples were irradiated with red light at 635 nm (100 mW/cm^2^) for 10 min. The decline in DPBF absorbance at 414 nm was then continuously monitored to determine the singlet oxygen generation efficiency of the samples.^42^

### Determination of Cytotoxicity

Cell viability was assessed using the (3-(4,5-dimethylthiazol-2yl)-2,5-diphenyltetrazolium bromide (MTT) assay. Briefly, 1 × 10^4^ cells per well were seeded into 96-well plates and allowed to adhere overnight. Subsequently, samples at six different concentrations prepared in growth medium were added to the wells. For the photocytotoxicity evaluation, cells were incubated with the samples for 4 h, after which the medium was removed, replaced with 1× PBS, and exposed to light (635 nm, 100 mW/cm^2^) for 3 min. For the dark cytotoxicity assessment (without light exposure), cells were similarly incubated with the samples for 4 h, followed by removal of the medium and washing with 1× PBS. In both assays, PBS was then discarded, and 100 μL of fresh growth medium was added. The plates were returned to the incubator for an additional 24 h incubation period. After this period, 30 μL of the MTT reagent was added under dark conditions and incubated at 37 °C for 3 h. The medium was then removed, and 100 μL of DMSO was added to dissolve the formed formazan crystals. Cell viability was determined by measuring absorbance using an ELISA reader (Synergy H1, BioTek).^42^

In all subsequent in vitro studies, the concentrations of free drugs and their corresponding amounts in nanoformulations were adjusted to 0.85 μM for PpIX and 2.5 μM for Dox. Samples without treatment were used as the control (CTRL) group throughout all experiments.

### Calcein AM/PI Staining

For the live/dead staining assay 4T1 cells were seeded at a density of 2 × 10^5^ cells per well in 24-well plates and allowed to adhere overnight. After treatment with the indicated NPs and free drugs for 24 h, the culture medium was removed, and the cells were gently washed twice with PBS. Subsequently, cells were incubated with a staining solution containing Calcein-AM (2 μM) and PI (5 μg/mL) prepared in serum-free medium for 20 min at 37 °C in the dark. After incubation, the staining solution was discarded, and the cells were washed twice with PBS to remove excess dye.

Fluorescence images were acquired by using a Zeiss Axio Observer Z1 microscope with Zeiss ZEN 2 imaging software. Live cells were visualized by calcein-AM staining (green fluorescence), while dead cells were identified by PI staining (red fluorescence). The percentage of live and dead cells was quantified by analyzing fluorescence images using ImageJ software, and the cytotoxic response was determined by counting the ratio of dead cells to live cells.^43^

### Determination of Cellular Uptake

The cellular uptake of NP-Dox@M1, PpIX, Dox, and Dox-PpIX was assessed using fluorescence microscopy and flow cytometry. 3T3-L1 and 4T1 cells were seeded at a density of 2 × 10^5^ cells per well in 24-well plates and allowed to adhere overnight. The samples were then added to the wells. Fluorescence microscopy images were captured using a Zeiss Axio Observer Z1 microscope with Zeiss ZEN 2 imaging software at predetermined time intervals (2, 4, and 24 h). Mean fluorescence intensity (MFI) measurements were performed as previously described.

The same experimental conditions were applied for flow cytometry, with incubation periods of 2, 4, and 24 h, and cells treated with only the medium served as a blank control. A total of 20,000 events were recorded per sample using Agilent NovoCyte Spectral Flow Cytometer Systems and analyzed with NovoExpress and FlowJo software. Each experiment was repeated at least three times.^42^

### Free Fructose Competition Assay

For competition assay 4T1 cells were seeded at a density of 2 × 10^5^ cells per well in 24-well plates, and then, the culture medium was replaced with serum-free medium, and cells were incubated overnight under serum-starvation conditions. To evaluate the effect of free fructose competition, cells were incubated with 2 mM free fructose for 1 h prior to treatment. Control groups were maintained under identical conditions without fructose supplementation. Following fructose preincubation, cells were treated with NP-Dox@M1, NP-Dox, or Dox + PpIX for either 2 or 4 h. At the end of the incubation period, cells were washed with phosphate-buffered saline (PBS) and stained with DAPI. Fluorescence images were subsequently acquired using a fluorescence microscope (Zeiss Axio Observer Z1 microscope). MFI measurements were performed as previously described.^44,45^

### Intracellular ROS Measurement

The levels of intracellular reactive oxygen species (ROS) in 3T3-L1 and 4T1 cells were evaluated using the 2′,7′-dichlorodihydrofluorescein diacetate (DCFH-DA) assay. 3T3-L1 and 4T1 cells (2.0 × 10^4^ cells per well) were seeded into 48-well plates and treated in accordance with the previously described protocol. After treatment, the cells were collected and incubated with 10 μM DCFH-DA in the dark for 30 min. DCFH-DA was hydrolyzed by intracellular esterases within the cells to form nonfluorescent DCFH, which was subsequently oxidized by ROS to produce the fluorescent compound DCF. The fluorescence intensity, which corresponds to ROS levels, was measured using Agilent NovoCyte Spectral Flow Cytometer Systems and analyzed using NovoExpress and FlowJo Software.^42^

### Cell Cycle Assay

3T3-L1 and 4T1 cells (2.0 × 10^4^ cells per well) were seeded in 48-well plates and incubated overnight to allow the cells to attach. The cells were then treated as previously described and prepared for cell cycle evaluation. After treatment, the cells were harvested, washed with PBS, and fixed in 70% ethanol at – 20 °C for 2 h. The fixed cells were then stained with propidium iodide (PI) and incubated with RNase to remove RNA. Cell cycle distribution was subsequently determined using flow cytometry, and the results were analyzed using NovoExpress software.^38^ The raw flow cytometry data are included in the Supporting Information.

### Apoptosis Assay

The effects of NP, NP@M1, NP-Dox, NP-Dox@M1, PpIX, Dox, and Dox-PpIX on apoptosis were evaluated using flow cytometry with and without light irradiation. The cells were exposed to compounds for 24 h. All of the compound dilutions and the control (CTRL) were prepared in cell culture medium. Following this, the cells were washed with a staining buffer and resuspended in binding buffer. FITC Annexin V and propidium iodide (PI) were then added to the cell suspension. The samples were left to incubate for 15 min in the dark before being analyzed using NovoExpress and FlowJo software.^46^ The raw flow cytometry data are provided in the Supporting Information.

### Apoptosis-Related Immunoblotting Assay

In the case of apoptosis-related immunoblotting tests, NPs and free drugs were applied to 4T1 cells as mentioned before, with or without light exposure, and finally, proteins were isolated in the RIPA buffer. HRP-conjugated GAPDH, 1:1000, Abcam; BCL-2, 1:1000, St John’s Lab, PARP 1:1000, CST; pro-caspase 3, 1:1000, CST; and pro-caspase 8, 1:1000, Abcam antibodies diluted in blocking solution were used as primary antibodies. Western blot analysis was performed as described above.

### Macrophage Uptake

The in vitro cellular uptake of NPs and free drugs in RAW264.7 macrophage cells was assessed by fluorescence microscopy. Briefly, macrophages were seeded in 12-well plates at a density of 4 × 10^5^ cells per well and allowed to adhere for 4 h. The formulations were then added and incubated for an additional 4 h. Following incubation, cells were stained with DAPI (1 μg/mL) for 15 min at 37 °C. Fluorescence images were acquired using a Zeiss Axio Observer Z1 microscope and processed with Zeiss ZEN2 imaging software, while mean fluorescence intensity (MFI) was determined as previously described.^47^

### Complementary Activation-Related Immunoblotting Assay

For complementary activation immunoblotting assays, proteins were extracted from NPs and free drugs-treated RAW 264.7 cells. First, cells were plated in 6-well plates at a ratio of 8 × 10^5^ wells/cell. They were incubated with NPs and free drugs for 4 h. After 4 h, the medium was removed, and fresh medium was added. After a total of 24 h, the cells were washed with 1x PBS, and proteins were extracted using RIPA buffer. p-STAT3 S727, 1:2000, abclonal; NF-κB p-p65, 1:1000, CST; and HRP-conjugated GAPDH, 1:1000, Abcam antibodies diluted in blocking solution were used as primary antibodies. Western blot analysis was performed as described above.

### Macrophage Polarization

The effect of NPs and free drugs on macrophage polarization was evaluated in RAW264.7 cells using flow cytometry to assess the presence of M1-type markers, CD80 and CD86. First, cells were plated in 12-well plates at a ratio of 4 × 10^5^ cells/well. They were incubated with NPs and free drugs for 4 h. After 4 h, the medium was removed, and fresh medium was added. After a total of 24 h, the cells were washed with 1x PBS and 1x cell staining buffer. Then, the cells were stained with FITC-conjugated CD80 and FITC-conjugated CD86 for 20 min at room temperature in the dark before being analyzed using NovoExpress and FlowJo software.^39^ The raw flow cytometry data are provided in the Supporting Information.

### Statistical Analysis

Statistical analyses were conducted using GraphPad Prism v10, employing one-way ANOVA analysis, followed by Tukey post hoc tests for multiple comparisons and a paired *t*-test for two-group comparisons. Data were expressed as means ± standard deviation (SD) with statistical significance set as at least *p* < 0.05. The significance levels were determined as *p ≤ 0.05, **p ≤ 0.01, ***p ≤ 0.001, and ****p ≤ 0.0001.

## Results and Discussion

### Biomimetic Hybrid Nanoplatform Synthesis

Biomimetic hybrid nanoplatforms are constructed through a stepwise workflow that synthesizes polymeric nanoplatforms endowed with GLUT5-targeting capability, and subsequently, cellular membranes of macrophages, which polarized toward a pro-inflammatory M1 phenotype, are harvested to generate vesicular membrane fractions, which are then fused with the cholesterol-functionalized glycopolymeric core via extrusion to yield hybrid nanostructures (Scheme 1).

**Scheme 1:**
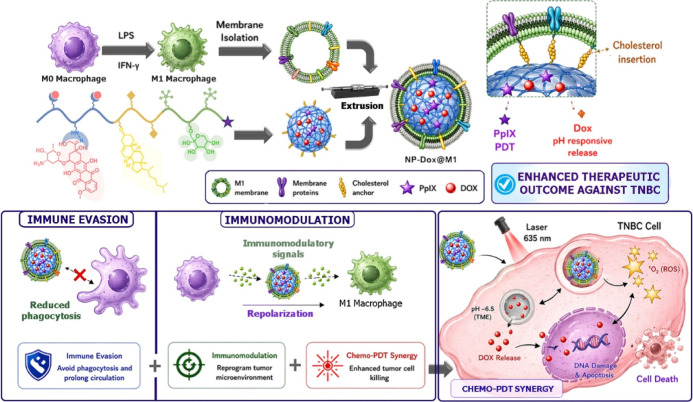
Illustration of the Cholesterol-Assisted, Membrane-Engineered Glycopolymer-Based Biomimetic Nanoplatform and Its Enhanced Therapeutic Outcomes against Breast Cancer

The polymer core of the biomimetic hybrid nanoplatform was synthesized via reversible addition–fragmentation chain-transfer (RAFT) polymerization. The precursor molecules were synthesized by following published procedures and characterized using ^1^H and ^13^C NMR spectroscopy (Figures S1 and S2). The cholesterol-functionalized glycopolymer, P­(*i*FMA-*b*-(CMA-*co-b*HMA)), was constructed the first from isopropylidene-protected fructose methacrylate (*i*FMA) and then by chain extension copolymerization of cholesterol methacrylate (CMA) and *tert*-butoxy carbonyl-protected hydrazine methacrylate (*b*HMA) monomers. ^1^H NMR spectra were acquired at each polymerization step to monitor the polymerization progress and quantify the incorporation of monomer units along the polymer chain. The ^1^H NMR spectrum of P­(*i*FMA) (P1) revealed that, based on the integration ratio between the seven protons of the sugar monomer (δ 3.5–5.0 ppm) and the phenyl protons of the RAFT agent (δ 7.86, 7.52, and 7.36 ppm), the polymer contained approximately 24 repeating sugar units (Figure S3A). The number-average molecular weight (*M*_n_) of P1, as determined by ^1^H NMR, was found to be 8.20 kDa, corresponding to a monomer conversion of 80%. According to GPC analysis, the *M*_n,GPC_ and polydispersity index (PDI) values of P1 were found to be 4.90 kDa and 1.10, respectively (Figure S3C, Table 1). Then, chain extension copolymerization of CMA and *b*HMA was performed. Through analysis of the ^1^H NMR spectrum of P­(*i*FMA-*b*-(CMA-*co*-*b*HMA)) (P2), it was determined that the polymer contained approximately 13 repeating units of CMA and 5 repeating units of *b*HMA, based on the integration ratios of the −*HC*=*C*– protons of CMA at δ 5.33 ppm and the −*N* H protons of *b*HMA at δ 6.56 and 10.05 ppm, compared to the phenyl protons of the RAFT agent (Figure S3B).

**Table 1:** Molecular Weight Characteristics of Polymers^a^

Polymer	[M]:[RAFT]:[I]	Con. (%)	*M*_n,NMR_ (kDa)	*M_n,GPC_ (kDa)	*M_w_/*M*_n_
P(*i*FMA)_24_ **(P1)**	[30]:[1]:[0.125]	80	8.20	4.90	1.10
P(*i*FMA_24_-*b*-(CMA_13_-*co*-*b*HMA_5_)) **(P2)**	[15–5]:[1]:[0.1]	>85	15.11	5.21	1.17
P(*i*FMA_24_-*b*-(CMA_13_-*co*-*b*HMA_5_))-PpIX **(P3)**	-	-	15.76	10.32	1.25
P(FMA_24_-*b*-(CMA_13_-*co*-HMA_5_))-PpIX **(P4)**	-	-	13.33	8.78	1.24
P(FMA_24_-*b*-(CMA_13_-*co*-HMA_5_-Dox))-PpIX **(P5)**	-	-	16.23	9.21	1.25

a The conversion was determined from ^1^H NMR results. Molecular weight (*M*_n_, kDa) and polydispersity index (*M*_w_/*M*_n_) were determined from DMF GPC-MALS results.

The *M*_n,NMR_ and monomer conversion of P2 were calculated from the ^1^H NMR spectrum (Figure S3) and were found to be 15.11 kDa and >85% monomer conversion, respectively. According to GPC analysis, the *M*_n,GPC_ and PDI values of P2 were also found to be 5.21 kDa and 1.17, respectively (Figure S3D, Table 1). Additionally, P1 and P2 were characterized by FT-IR spectroscopy. The FT-IR spectrum for P1 exhibited C–H stretching vibration peaks of the sugar backbone at 2920–2850 cm^–1^. A distinct carbonyl (C=O) stretching band appeared at 1750 cm^–1^, which is consistent with the ester functionality of the methacrylate units. In the FT-IR spectrum for P2, the intensity of the carbonyl stretching band at 1750 cm^–1^ increased, indicating the successful incorporation of the CMA and *b*HMA units. The C–H stretching region (∼2850–2950 cm^–1^) shifted and broadened to reflect the aliphatic and sterol moieties characteristic of cholesterol. Furthermore, a new N–H stretching band of the *b*HMA units was observed at ∼3250 cm^–1^, confirming the presence of amide functionalities. Both FTIR spectra exhibited a distinct absorption band at around 2100 cm^–1^, which is characteristic of alkyne functionality (Figure S3E). The presence of the alkyne group on the polymer chain end was confirmed by ^1^H NMR spectroscopy, which revealed a corresponding proton signal at δ 2.5 ppm, consistent with the terminal alkyne moiety.

Subsequently, azide-functionalized PpIX was conjugated to the polymer backbone via a CuAAC click reaction. The successful attachment was confirmed by ^1^H NMR spectroscopy, which revealed the appearance of characteristic signals corresponding to the triazole linkage and the PpIX moiety (Figure S4A). GPC results showed that P3 exhibited a narrow molecular weight distribution with an *M*_n,GPC_ and PDI of 10.32 kDa and 1.25, respectively (Table 1).

Following TFA-mediated hydrolysis, the isopropylidene (*i*) and *tert*-butoxycarbonyl (*b*) protecting groups were successfully removed from the polymer backbone. This was confirmed by ^1^H NMR spectroscopy, which revealed the disappearance of the characteristic signals corresponding to the isopropylidene methyl protons and *tert*-butyl groups (Figure S4B). Additionally, GPC results indicated that P4 had a unimodal molecular weight distribution with an *M*_n,GPC_ of 8.78 kDa and a PDI of 1.24 (Table 1). Also, the regeneration of the functional groups was also evident from the appearance of new signals in the FT-IR spectrum. The success of the hydrolysis reaction was confirmed by the disappearance of isopropylidene and *tert*-butyl and the appearance of hydroxyl and amino groups of FMA and HMA units via FT-IR (Figure S5A)^48,49^ . In addition, UV–vis spectroscopy provided further evidence of the incorporation of PpIX, as demonstrated by the presence of its characteristic Soret band at ∼400–410 nm and its Q-bands at 500–650 nm in the P4 spectrum (Figure S5B).

Figure S6 shows the degradation rate of DPBF upon irradiation in the presence of P4 over the time range of 0–10 min. The singlet oxygen quantum yield (Φ_Δ_) of P4 was calculated from the DPBF photodegradation rates obtained under identical experimental conditions using PpIX as the reference photosensitizer.^50,51^ The detailed experimental procedure and the corresponding equation are provided in the Supporting Information. The results show that P4 exhibits a high singlet oxygen generation efficiency with a Φ_Δ_ value of 0.69, indicating efficient singlet oxygen production upon 635 nm light irradiation.

The conjugation of Dox was verified by ^1^H NMR spectroscopy, as indicated by the emergence of characteristic signals attributed to the aromatic *–O* H protons of Dox at δ 14.31 and δ 13.44 ppm. The extent of Dox coupling was quantitatively determined through integration analysis by comparing the signal areas of the two *–C* H=*CH*_*2*_ protons of PpIX (δ 8.54 ppm) and the *–N* H protons of HMA repeating units at δ 10.35 ppm with those of the aromatic *–O* H protons of Dox. Five Dox units were conjugated to the glycopolymer, and the conjugation efficiency was determined to be greater than 99% based on ^1^H NMR integration data (Figure S4C). Also, Dox loading content and conjugation efficiency determined by UV–vis analysis were found to be ∼0.165 mg/mL, consistent with the values obtained from ^1^H NMR analysis (0.167 mg/mL), confirming the successful incorporation of Dox into the polymer backbone. The GPC results revealed that P5 exhibited an *M*_n,GPC_ of 9.21 kDa and a PDI of 1.25, indicating a relatively uniform molecular weight distribution and suggesting good control over the conjugation process (Table 1). Following the conjugation of Dox, the FT-IR spectrum of P5 exhibited a broad absorption band around 3550 cm^–1^, which was attributed to the overlapping stretching vibrations of the NH_2_ groups of Dox and the O–H groups. In addition, the characteristic C=N stretching band of the newly formed hydrazone linkage was observed at approximately 1650 cm^–1^, in good agreement with the previously reported literature.^52,53^ According to the FT-IR results, the successful attachment of Dox is confirmed (Figure S5A). Also, for the determination of PpIX and Dox amount within the polymers, calibration curves were generated using UV–vis spectroscopy (Figure S5B,C).

For the preparation of cell membranes, RAW264.7 macrophages were first polarized toward the M1 phenotype with LPS and IFNγ treatment prior to membrane isolation. M1-type polarization of macrophage cells was assessed by flow cytometry using M1-type markers CD80 and CD86 (Figure S7). The polarized cells were then subjected to hypotonic lysis, followed by ultrasonic disruption to facilitate membrane release, and intracellular components were subsequently removed through density gradient centrifugation. The total protein content of the prepared vesicles was quantified using a bicinchoninic acid (BCA) assay for SDS PAGE analysis.

Biomimetic hybrid nanostructures were subsequently fabricated by combining purified membrane fractions (M1) and the cholesterol-functionalized glycopolymeric core through ultrasonic mixing, followed by repeated extrusion to promote efficient membrane fusion and uniform vesicle formation using a nanoextrusion system, passing sequentially through 400 and 200 nm membranes. Systematic optimization of extrusion parameters, including particle size distribution, ζ-potential, and polydispersity index (PDI), identified 11 extrusion cycles as the optimal conditions to achieve highly uniform nanostructures (NP-Dox@M1) (Figure 1). Moreover, membrane-coated formulations without Dox (NP@M1) and membrane-uncoated formulations with (NP-Dox) or without Dox (NP) were also prepared to investigate the synergistic effect of dual therapy together with both enhanced cellular uptake and immunomodulation.

**Figure 1:**
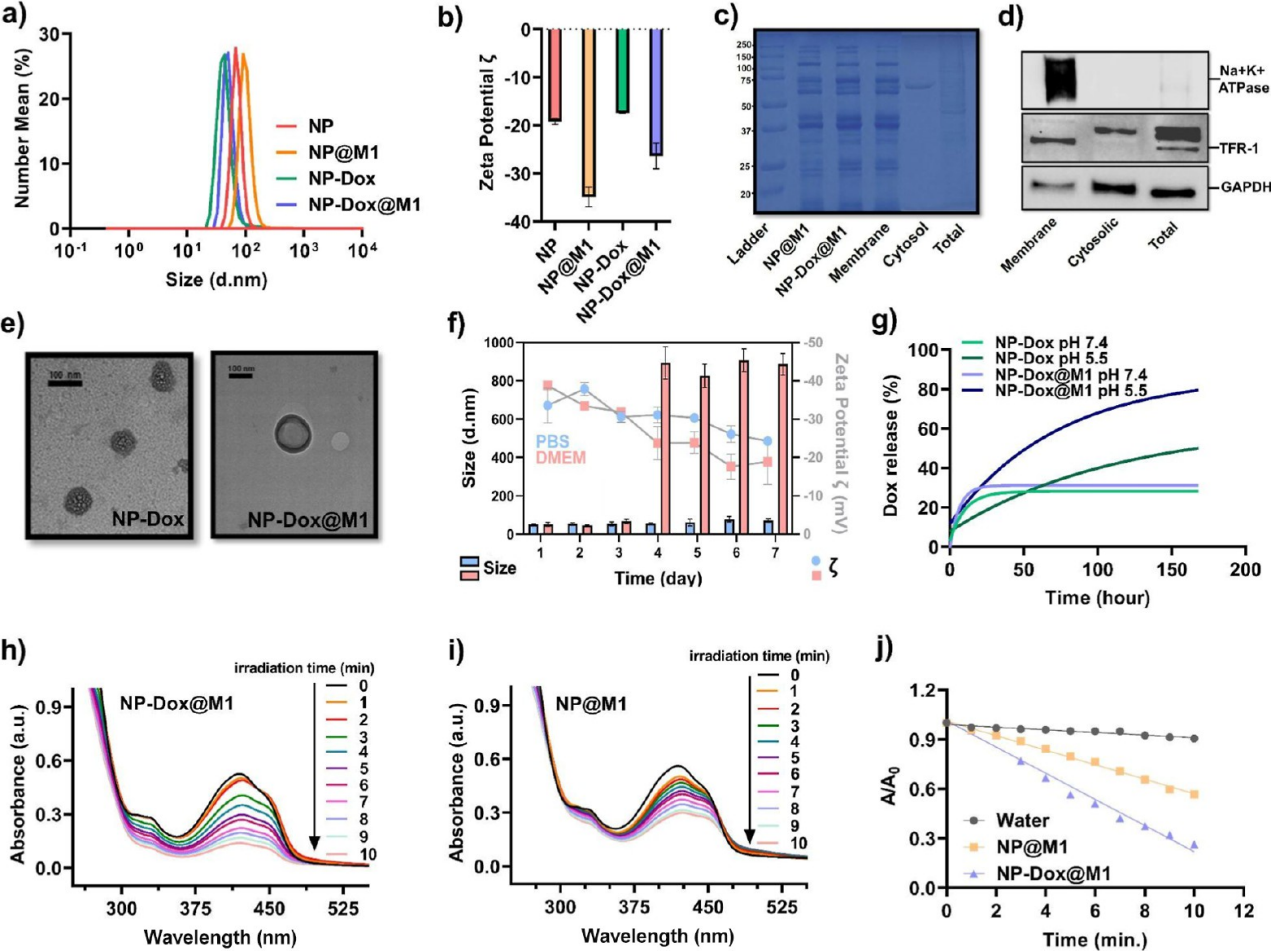
(a,b) DLS and zeta potential values of nanoplatforms, (c) SDS-PAGE analysis of NPs and cell proteins, (d) Western blot analysis of cell proteins, (e) TEM images of NP-Dox and NP-Dox@M1, scale bar: 100 nm, and (f) stability evaluation of NP-Dox@M1 at PBS (pH 7.4) and complete cell culture medium for 7 days; bars indicate the size (left axis), while symbols indicate the zeta potential (right axis). (g) Drug release kinetics of NP-Dox and NP-Dox@M1 at pH 7.4 and 5.5. (h,i) Time-dependent DPBF quenching curve indicating reactive oxygen species (ROS) generation by NP-Dox@M1 and NP@M1 under light irradiation. (j) The ratio (*A*/*A*_0_) of the singlet oxygen absorber DPBF initial absorbance to the absorbance values after stimulation with different durations.

Dynamic light scattering (DLS) analysis revealed that the uncoated NPs exhibited a mean hydrodynamic diameter of approximately 72 and 46 nm with a surface charge between −17 and −19 mV for NP and NP-Dox, respectively (Figure 1A,B). After membrane coating of NPs, the mean hydrodynamic diameter reached approximately 101 and 52 nm with a surface charge between −26 and −35 mV for NP@M1 and NP-Dox@M1, respectively. This size reduction originates from structural reorganization of the polymer assemblies after covalent attachment of Dox through hydrazone linkages. Doxorubicin contains multiple aromatic domains that can promote intermolecular hydrophobic interactions and π–π stacking within the nanoplatform core. Such interactions may lead to a more compact packing of the polymer chains and consequently a reduced hydrodynamic diameter.^54,55^

The protein expression profiles were used to confirm the successful coating of the nanoplatforms with the M1 macrophage membrane. The preservation and integrity of membrane-associated proteins were evaluated by using SDS-PAGE, which confirmed effective transfer of membrane components onto the nanoparticle surface. The patterns of protein distribution of the original macrophage membrane were also preserved in NP@M1 and NP-Dox@M1, as analyzed through SDS-PAGE analysis. Protein profiles of NP@M1 and NP-Dox@M1 were similar to those of the original cell membrane of the macrophage, indicating that the membrane proteins were efficiently retained during the isolation and coating processes (Figure 1C).

Western blot analysis was further carried out to verify the successful isolation and preservation of macrophage cell membranes. The results demonstrated the presence of Na^+^/K^+^-ATPase, an electrogenic transmembrane protein localized in the plasma membrane, and TFR-1, a transmembrane glycoprotein involved in transferrin binding and predominantly expressed on the cell surface. Chemiluminescent detection further confirmed that membrane-associated proteins were present in the isolated membrane protein lysate, whereas they were absent in the cytosolic fraction (Figure 1D).

Morphological characterization by transmission electron microscopy (TEM) of negatively stained samples NP-Dox and NP-Dox@M1 confirmed the presence of well-defined spherical vesicles with a characteristic bilayer structure (Figure 1E). NP-Dox takes the shape of discrete and well-dispersed spherical particles of relatively uniform size distribution with dimensions of approximately 50 nm, yielding results that are also compatible with DLS. The particles have a solid and dense core and sharp boundaries, which reflect the success of the formation of nanoparticles and the integrity of their structure. Conversely, NP-Dox@M1 shows a significant structural change after coating with the cell membrane. The nanoplatforms retain their general morphology of a sphere; a clear core–shell structure emerges. In particular, the darker inner core is covered by a lighter, more diffuse outer layer, which can be attributed to the presence of the cell membrane coating. This shell-like structure signifies that the polymeric nanoplatform was successfully encapsulated by the membrane.

Notably, stability studies demonstrated that NP-Dox@M1 retained its particle size and ζ-potential with minimal variation over a period of at least 7 days in both PBS (Figure 1F). Furthermore, while NP-Dox@M1 remained stable in terms of size and ζ-potential for 3 days in complete cell culture medium, the size and ζ-potential values of the nanoplatform differed after this time. A significant increase in hydrodynamic diameter and a shift toward a more positive ζ-potential were observed in the complete cell culture medium at the end of 1 week, suggesting the formation of a protein corona on the particle surface, likely driven by the adsorption of serum proteins.

### Drug Release

The release behavior of NP-Dox and NP-Dox@M1 in vitro under physiological (pH 7.4) and acidic (pH 5.5) conditions and a maximum of 168 h was examined to understand the release behavior of the two materials in response to pH (Figure 1G). Both formulations exhibited a time-dependent release pattern, while the release rate was markedly accelerated under acidic conditions. At pH 7.4, the uncoated nanoplatform demonstrated a relatively slow and sustained release, reaching 38.23% cumulative release at 168 h. Similarly, the membrane-coated nanoplatform showed a controlled release behavior at pH 7.4, reaching 36.89% at 168 h, indicating that the membrane coating did not significantly increase drug leakage under physiological conditions. Both NP-Dox and NP-Dox@M1 exhibited a slow and sustained release, suggesting that the polymeric matrix and the membrane layer remained structurally stable and effectively prevented premature drug leakage during the circulation.

In contrast, both formulations recorded significantly improved drug release at pH 5.5. The NP-Dox cumulative release was 52.44% at 168 h, and NP-Dox@M1 had a significantly higher release profile of 81.53%. This strong rise in drug release under acidic conditions indicates that more efficient payload release in tumor/endosomal-like environments is achieved by using the membrane-engineered formulation. This increased release at pH 5.5 can be explained by the destabilization of the nanoplatform structure, such as partial membrane destabilization of the macrophage and destabilization of the hydrophobic interaction between the membrane and the cholesterol-based glycopolymeric core, which enables greater permeability and diffusion of drugs. Taken together, these findings verify that NP-Dox@M1 can be kept stable at physiological pH and can release more under acidic conditions, which supports its potential use as a tumor-selective drug carrier and enhanced clinical outcome.

### Singlet Oxygen Generation

The reactive oxygen species (ROS) generation capability of NP@M1 and NP-Dox@M1 was evaluated using a DPBF photodegradation assay under light irradiation (635 nm, 100 mW/cm^2^) for 10 min (Figure 1H–J). ROS generation was quantified by monitoring the decrease in DPBF absorbance expressed as the *A*/*A*_0_ ratio over time.

As shown by the time-dependent reduction in DPBF absorbance, the water group exhibited minimal DPBF degradation, with the *A*/*A*_0_ value remaining relatively stable throughout irradiation, confirming negligible ROS generation in the absence of a photosensitizer. In contrast, both the NP@M1 and NP-Dox@M1 groups induced a pronounced decrease in DPBF absorbance upon laser exposure, demonstrating efficient ROS production. The NP@M1 formulation showed a gradual reduction in *A*/*A*_0_, reaching 0.567 at 10 min. Notably, NP-Dox@M1 exhibited the most significant DPBF degradation, with the *A*/*A*_0_ ratio decreasing to 0.265 at 10 min, indicating markedly enhanced ROS generation approximately 2.14 times higher than that of the NP@M1 group. The enhanced ROS generation observed in the NP-Dox@M1 group may be attributed to the coloading of Dox, which could improve the overall photoreactivity of the system by promoting intraparticle interactions and facilitating more efficient energy transfer and photosensitizer activation under laser irradiation. These results confirm that the NP-Dox@M1 formulation displays the highest photodynamic activity, supporting its potential for enhanced PDT-mediated anticancer therapy.

### *In Vitro* Studies

#### Cytotoxicity

To evaluate the cell-specific cytotoxicity of the nanoplatforms, 3T3-L1- and GLUT5-overexpressing 4T1 cell lines were used in cell viability assays (Figures S8, 2A). The IC_50_ values were assessed to determine phototoxicity and the potential for therapeutic use of the biomimetic nanoplatforms. The cytotoxic effects of all nanoplatforms, as well as free mono- and dual-drug combinations, were assessed using the MTT assay with and without light exposure. The results demonstrated that biomimetic nanoplatforms significantly reduced the proliferation of breast cancer cells compared to free mono/dual drug combinations and mono-/dual-modal nanoplatforms in the absence of light irradiation. As expected, owing to the photosensitive nature of PpIX, these biomimetic nanoplatforms exhibited enhanced efficacy and increased cytotoxicity in 4T1 cells under light exposure compared to free drug formulations. In particular, NP-Dox and NP-Dox@M1 groups showed a synergistic cytotoxic effect on 4T1 cells when combined with PpIX under light irradiation. IC_50_ analysis further confirmed that NP-Dox@M1 exhibited the highest level of toxicity in 4T1 cells. It is important to note that the nanoplexes mainly differ in their outer surface coatings. A possible explanation for the enhanced effect is the overexpression of GLUT transporters in cancer cells, which likely facilitates stronger interactions with fructose moieties on the nanoplatform surface, thereby improving cellular uptake and cytotoxicity. Additionally, cell viability remained within safe and appropriate limits for all nanoplatforms containing 0.85 μM PpIX and 2.5 μM Dox, regardless of light exposure. These concentrations were therefore considered safe and suitable for subsequent biological experiments.

**Figure 2:**
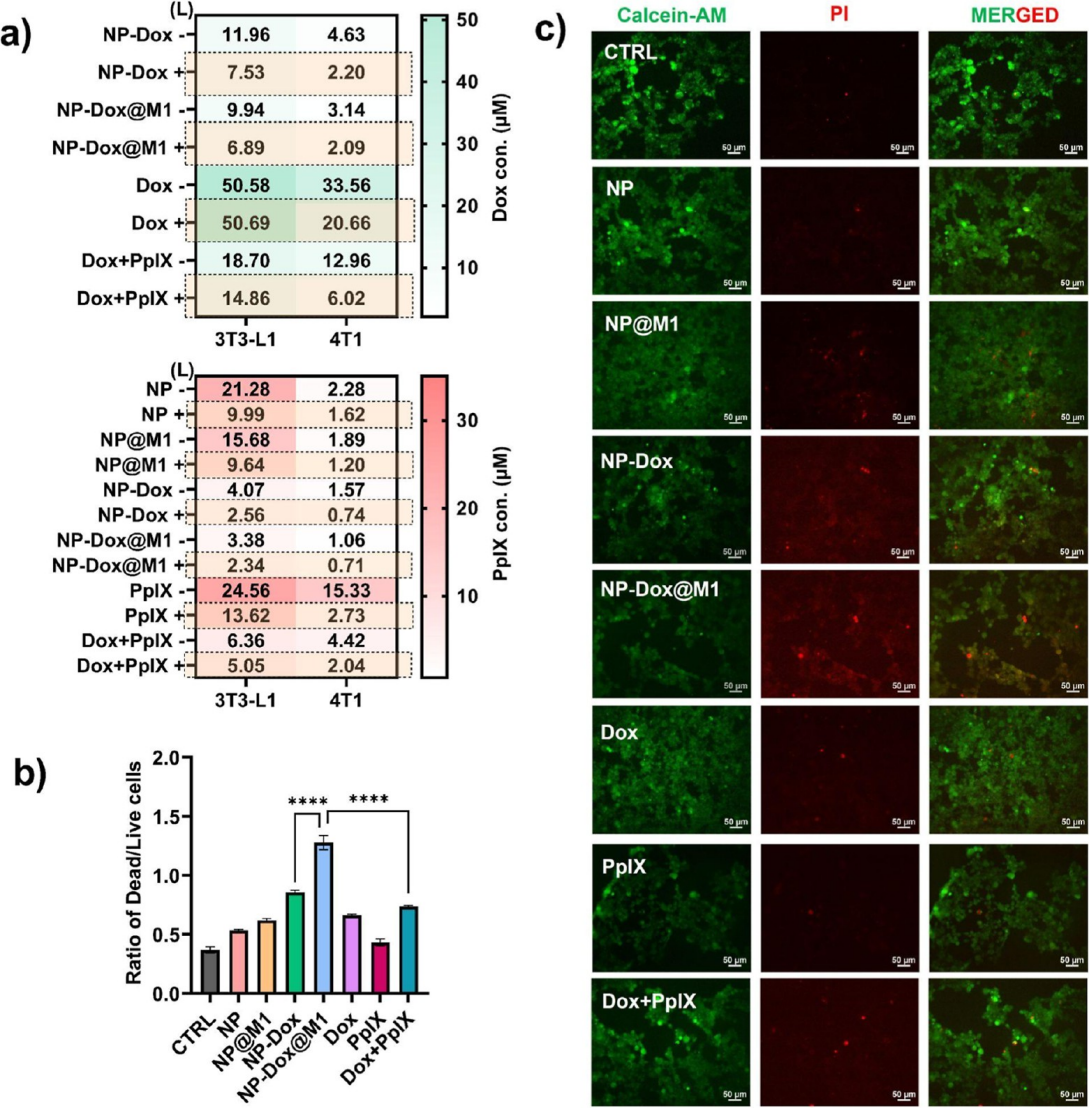
(a) IC_50_ values of NPs and free drugs in 3T3-L1 and 4T1 cell lines based on PpIX and Dox concentrations for 24 h. (b) The dead/live cell ratios of NPs and free drugs-treated 4T1 cells for 24 h according to Calcein AM/PI staining. (c) Fluorescence microscopy images of 4T1 cells stained with Calcein AM/PI after 24 h treatment with NPs and free drugs. Data are presented as means ± SD; ****p ≤ 0.0001.

The nanoplatform had moderate cytotoxicity under light irradiation conditions, which is mainly due to the chemotherapeutic payload. Comparatively, antiproliferative activities of free drug formulations and noncoated nanoplatforms were relatively less or similar, suggesting the role of the biomimetic M1 membrane coating in increasing cellular uptake and therapeutic activity. When cells were irradiated, cell viability significantly decreased in the nanoplatform-treated samples as compared to all nonirradiated counterparts. This increased cytotoxicity is because it activates the photosensitizer, resulting in the generation of reactive oxygen species (ROS) and synergy with a chemotherapeutic agent. It is worth noting that the biomimetic nanoplatform exhibited the greatest cytotoxicity of all groups under light irradiation conditions, which indicated the synergistic benefits of biomimetic targeting, cellular interaction through the glycopolymer, and dual-modal therapy. On the whole, these findings ensure that light-mediated activation greatly contributes to the effectiveness of the nanoplatform in the treatment of cancer, and the M1 membrane coating additionally advances the selective cytotoxicity of the system, identifying it as an efficient candidate for treating cancer in targeted therapy.

#### Calcein AM/PI Staining

The cytotoxicity of the developed nanoplatforms and free drugs was also assessed using Calcein AM/PI live–dead staining after 24 h of incubation with 4T1 cells (Figure 2B,C). The fluorescence microscopy images were obtained to observe Calcein AM positive viable and PI positive dead cells, and the cytotoxic response was determined by counting the ratio of dead cells to live cells.

The untreated group exhibited predominantly Calcein AM-positive cells with minimal PI staining, confirming a high cell viability under normal conditions. Similarly, the control formulations without Dox exhibited a comparatively low dead/live cell ratio, which demonstrated low cytotoxicity in the absence of a chemotherapeutic payload. In contrast, free Dox + PpIX treatment resulted in an increased proportion of PI-positive cells, while their combination further elevated the dead-to-live cell ratio, demonstrating enhanced cytotoxic activity.

The M1 macrophage membrane-coated nanoplatform that was coloaded with Dox and PpIX induced the highest cell death, as shown by a much higher dead/live cell ratio than the uncoated nanoplatform and the free Dox + PpIX mixture. These findings indicated that the therapeutic efficiency of the codelivery system with membrane camouflage was significantly enhanced, resulting in an increase in the cytotoxic outcome at 24 h exposure.

The Calcein AM/PI staining test indicated that the membrane-coated Dox/PpIX-loaded nanoplatform outperformed its noncoated counterpart and free drugs in the treatment performance, indicating that the membrane functionalization of macrophages was beneficial in enhancing the performance of nanoplatforms.

#### Cellular Uptake

The cellular uptake of the M1 membrane-coated biomimetic nanoplatforms was studied at 2, 4, and 24 h by flow cytometry and compared to that of free drug formulations and noncoated nanoplatforms (Figure 3A,B). The analysis of the flow cytometry showed that 4T1 cells exhibited an increase in cellular internalization with time in all nanoplatform groups, whereas the extent of uptake was comparatively low in all groups in the case of 3T3-L1 cells. At 4h, NP-Dox@M1 already exhibited a greater fluorescence intensity than free drug formulations, which implies faster cellular interaction and internalization.

**Figure 3:**
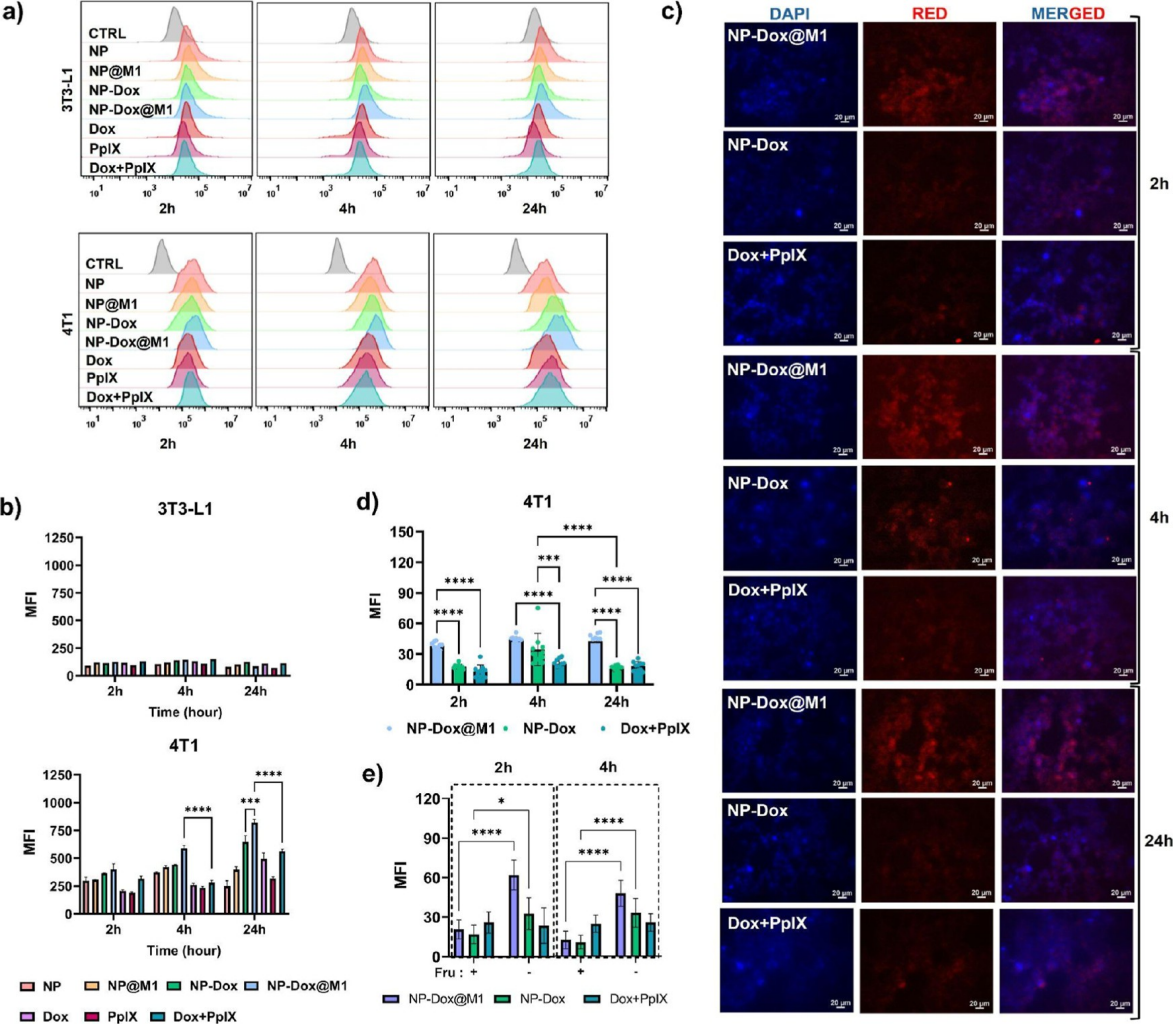
(a) Flow cytometric analysis after 2, 4, and 24 h treatment with NPs and free drugs of 3T3-L1 and 4T1 cells. (b) MFI values obtained from flow cytometry analysis of NPs and free drugs-treated 3T3-L1 and 4T1 cells for 2, 4, and 24 h. (c) Fluorescence microscopy images after 2, 4, and 24 h treatment with NPs and free drugs, indicating the cellular uptake of 4T1 cells. (d) MFI values obtained from fluorescence microscopy analysis of NPs and free drugs-treated 4T1 cells for 2, 4, and 24 h. (e) MFI values obtained from fluorescence microscopy analysis of NP-Dox@M1-, NP-Dox-, and Dox + PpIX-treated 4T1 cells after 2 and 4 h preincubated with or without free fructose (Fru+ and Fru-). Data are presented as means ± SD; *p ≤ 0.05, ***p ≤ 0.001, and ****p ≤ 0.0001.

At 4 h, the uptake of the nanoplatform-treated cells increased significantly, on one hand, due to the application of the nanoplatform and, on the other hand, due to the application of the M1-coated group, indicating the increased recognition and endocytosis of membranes. Conversely, there was a weak intracellular uptake in free drug combinations, which was probably because of passive diffusion and efflux.

The optimum cellular uptake was observed in the cells treated with NP-Dox@M1 at 24 h. This finding implies that the process of internalization is both long-term and efficient and, probably, supported by the interaction of the biomimetic membrane coating and fructose moieties with overexpressed GLUT transporters. There was moderate uptake of uncoated nanoplatforms, but the drug groups in the free form were much lower.

Then, the cellular uptake behavior of NP-Dox, NP-Dox@M1, and Dox + PpIX was also studied using fluorescence microscopy in 4T1 cells after incubating them with the nanoparticles after 2, 4, and 24 h (Figure 3C,D). The efficiency of uptake was compared by intracellular fluorescence intensity in the red channel of the Dox + PpIX-loaded nanoplatforms and free drugs.

NP-Dox@M1 had a much higher intracellular fluorescence signal than the uncoated nanoplatform and the free drug combination after 2 h of incubation, which indicates an improved early-stage cellular internalization. The nanoplatform covered with a membrane exhibited the highest uptake efficiency, and a much higher level of fluorescence was observed at 4 h than in the free Dox + PpIX group. The difference between the coated and uncoated nanoplatforms was statistically significant, but the significance was relatively smaller than during other time periods, which indicated a partial convergence of the intracellular accumulation over time, indicating that nanoplatforms are best at achieving cellular uptake after the fourth hour.

Importantly, the nanoplatform coated with the membrane at 24 h showed substantially higher intracellular accumulation compared to the uncoated nanoplatform and free drugs, confirming that it had better uptake behavior at longer incubation periods. Moreover, NP-Dox showed a time-dependent uptake profile, with the intracellular intensity level of the fluorescence at 4 h being much higher than the level of the fluorescence at 24 h, indicating less intracellular retention or exocytosis with longer incubation times.

These results indicate that the coating of membranes on macrophages greatly increases cellular uptake of NP-Dox@M1 in 4T1 cells, especially at early and late times, and validates that membrane functionalization is an effective method of intracellular delivery.

To investigate GLUT-5-mediated uptake of glycopolymeric biomimetic nanoplatforms, a fructose competition assay was performed, and the mean fluorescence intensity (MFI) values were determined after 2 and 4 h incubation periods (Figure S9, Figure 3e). The presence of fructose significantly reduced the cellular uptake of NP-Dox@M1, as evidenced by a marked decrease in MFI compared with that for the fructose-free condition at the end of 2 h (*p* < 0.0001). Similarly, NP-Dox exhibited a reduction in uptake in the presence of fructose preincubation (*p* < 0.05), whereas the MFI value of Dox + PpIX remained almost close in both conditions. Among all groups, NP-Dox@M1 showed the highest fluorescence intensity in the absence of fructose, indicating superior cellular internalization. A similar trend was observed after 4 h of incubation. The addition of fructose significantly decreased the uptake of NP-Dox@M1 and NP-Dox (*p* < 0.0001). In contrast, the uptake of Dox + PpIX was only minimally affected by fructose preincubation, with MFI values remaining relatively similar between fructose-treated and untreated conditions.

#### Intracellular ROS Measurement

The level of intracellular reactive oxygen species (ROS) was measured using the DCFDA fluorescence probe under both nonirradiated and light-irradiated conditions to study the photodynamic activity and oxidative stress potential of biomimetic nanoplatforms (Figure 4A-B). As expected, the untreated control group showed very little DCF fluorescence, indicating low baseline ROS. Without light, cells treated with PpIX also produced minimal ROS, confirming that ROS generation mainly depends on light. However, groups that combined Dox and PpIX showed a moderate increase in DCF fluorescence, likely due to drug-induced oxidative stress and changes in the redox balance.

**Figure 4:**
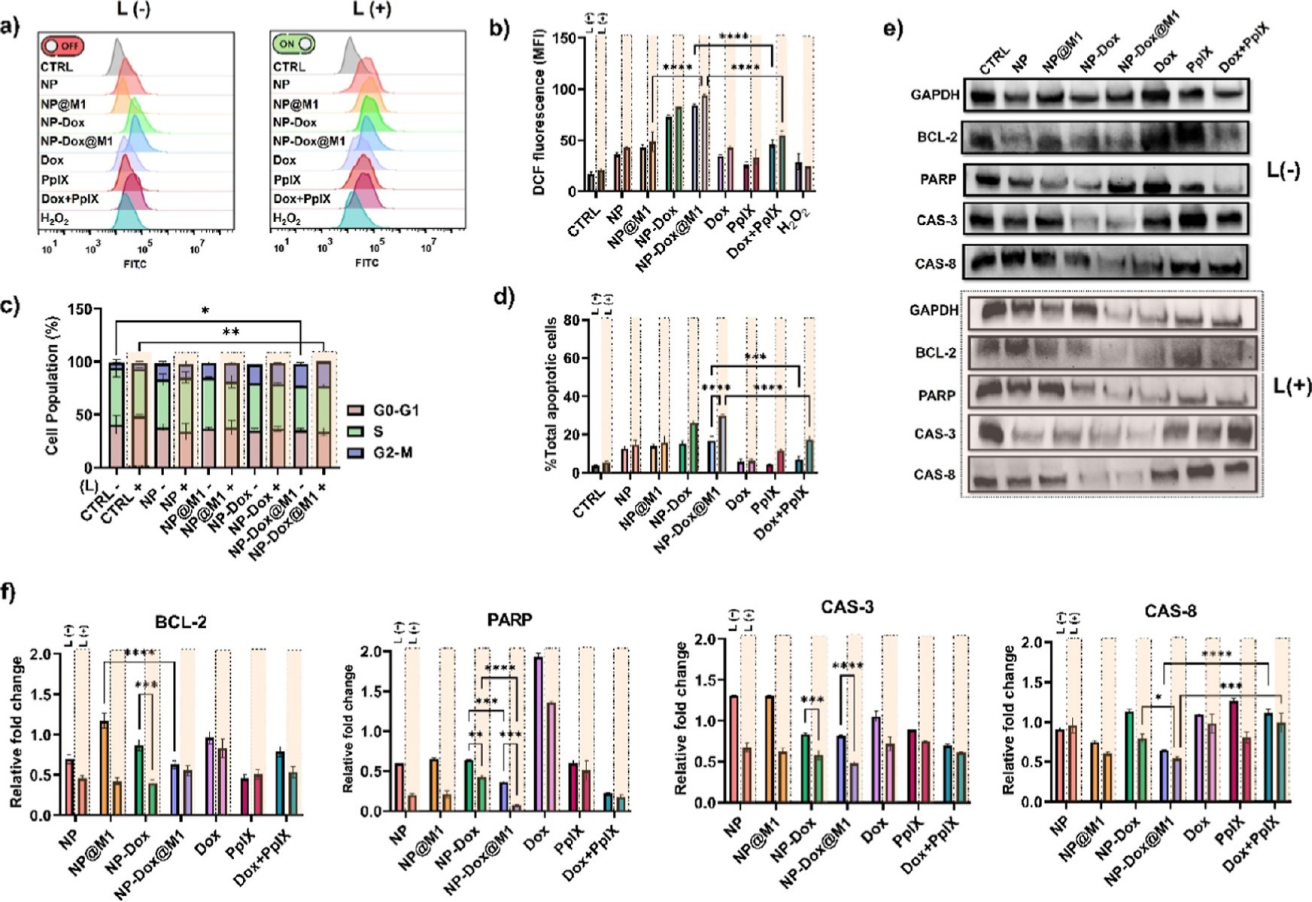
(a) Flow cytometry analysis of NPs- and free drugs-treated 4T1 cells stained with a DCFDA probe for ROS. (b) Intracellular ROS levels expressed as MFI in 4T1 cells by flow cytometry. (c) Cell cycle distribution of 4T1 cells after NPs and free drugs treatments. (d) Total apoptotic cell population of 4T1 cells (%) treated with NPs and free drugs. (e) Representative immunoblots of indicated proteins with GAPDH as a loading control (f) Densitometric quantification of apoptosis-related proteins normalized to GAPDH and expressed relative to CTRL. Pale yellow indicates light irradiation. Data are presented as means ± SD; *p ≤ 0.05, **p ≤ 0.01, ***p ≤ 0.001, and ****p ≤ 0.0001.

Upon light irradiation, a pronounced increase in DCF fluorescence intensity was detected in all PpIX-containing groups, confirming efficient photodynamic activation and subsequent ROS production. Furthermore, M1 macrophage membrane-coated nanoplatforms produced the highest ROS signal under irradiation, indicating that biomimetic membrane functionalization effectively enhanced the intracellular delivery and photodynamic efficiency. Notably, NP@M1 and NP-Dox@M1 exhibited significantly higher ROS levels than free PpIX and Dox + PpIX, suggesting enhanced cellular uptake and improved intracellular retention of the photodynamic agent.

The combined chemo-photodynamic nanoplatform NP-Dox@M1 led to more ROS than single-agent treatments, suggesting a possible synergy between chemotherapy-induced and photodynamically generated ROS. These results show that the M1 macrophage-membrane-coated biomimetic nanoplatform can boost and control ROS generation, supporting its promise as an effective light-activated therapy.

#### Cell Cycle

Cell cycle progression was evaluated by propidium iodide (PI)/RNase staining, followed by flow cytometry to investigate the antiproliferative effects of the developed biomimetic nanoplatforms and to elucidate their influence on cell cycle regulation (Figures S10 and 4C). The untreated control group displayed a typical cell cycle distribution, with the majority of cells residing in the G0/G1 phase, followed by cells in the S and G2/M phases, indicating normal proliferative behavior.

Under nonirradiated conditions, treatment with NPs and NP@M1 did not result in a significant alteration in cell cycle distribution compared to the control group, confirming negligible dark toxicity. In contrast, NP-Dox@M1 exhibited a clear perturbation of the cell cycle profile. Specifically, an increased accumulation of cells in the G2/M phase was observed, which is consistent with the well-established mechanism of Dox involving DNA intercalation, leading to DNA damage and activation of cell cycle checkpoints. This effect was more pronounced in the Dox-containing biomimetic nanoplatform, suggesting improved intracellular delivery and enhanced therapeutic activity.

Following light irradiation, nanoplatforms demonstrated significantly enhanced cell cycle disruption relative to their nonirradiated counterparts. A marked enrichment of cells in the G2/M phase was detected, suggesting that photodynamically generated ROS further intensified Dox-mediated genotoxic stress, thereby enhancing checkpoint activation and inhibiting cell cycle progression.

Among all of the treatment groups, the M1 macrophage membrane-coated nanoplatform exhibited the strongest impact on cell cycle distribution upon irradiation, resulting in the most pronounced G2/M arrest. This enhanced effect can be attributed to the biomimetic membrane coating, which likely improves the cellular internalization and increases the intracellular accumulation of both Dox and PpIX. These findings demonstrate that the M1 macrophage membrane-coated nanoplatform effectively induces Dox-associated cell cycle arrest, particularly under light stimulation, supporting its potential as a highly effective combinational chemo-photodynamic therapeutic system.

#### Apoptosis

Quantification of apoptotic and necrotic cell populations was performed using the Annexin V/7-AAD dual stain and flow cytometry to determine the cytotoxic efficacy of the developed nanoplatforms in the presence of no irradiation and light irradiation (Figures S11, 4D). Most cells were still alive (Annexin V-/7-AAD-) in the untreated control group, which showed little basal apoptosis. Equally, the treatment of cells with free PpIX under the nonirradiated conditions showed an equal viability profile with the control group, indicating that there was negligible dark toxicity.

By contrast, the use of Dox-containing formulations NP-Dox and NP-Dox@M1 led to a significant increase in the populations of apoptotic cells despite the lack of light stimulation. In particular, it was found that the number of apoptotic cells had increased, and this is in line with the established sequence of Dox-induced DNA damage that causes apoptosis.

After light exposure, biomimetic nanoplatforms exhibited a significant change in viable populations to apoptotic populations and showed effective PDT-mediated cytotoxicity, particularly in NP-Dox@M1. It is interesting to note that the chemo-photodynamic nanoplatform was the most effective with the highest apoptotic response in terms of significant enhancement in the total apoptotic fractions. This increased effect indicates that there is a synergistic interaction between ROS generated by PDT and Dox-induced genotoxic stress, leading to an amplified apoptotic signaling.

The M1 macrophage membrane-coated nanoplatform was found to be the most apoptotic on irradiation with a great percentage of reduction in viable cells and the highest percentage of annexin V-positive populations. This observation shows that functionalization of biomimetic membranes enhances intracellular delivery and therapeutic accumulation of both Dox and PpIX, thus enhancing the overall cytotoxic effect. Taken together, these findings indicate that the M1 membrane-coated biomimetic nanoplatform is a promising apoptosis-inducing and therapeutic delivery system especially when activated by light, which makes it a promising combinational chemo-photodynamic therapeutic platform.

#### Apoptosis-Related Immunoblotting Assay

As an extension of the study of the apoptosis-associated molecular mechanisms triggered by the developed nanoplatforms, Western blot analysis was used to assess the levels of expression of BCL-2, total PARP, procaspase-3, and procaspase-8 (Figure 4E,F). The loading control was GAPDH, so that a semiquantitative comparison was done using the normalized band intensities against GAPDH, and the fold change was given as relative to untreated control.

Western blot analysis under nonirradiated conditions showed that the levels of BCL-2 protein and total protein amounts of PARP in the NP-Dox and NP-Dox@M1 groups are significantly lower than those in the control groups. This reduction shows that Dox treatment was adequate to inhibit antiapoptotic signaling and induce apoptosis-associated molecular changes, which confirms the cytotoxic and pro-apoptotic effect of Dox even without light stimulation. Free nanoplatforms and Dox-free nanoplatforms had comparatively high pro-caspase-3 and pro-caspase-8 expression, meaning that there was little activation of apoptotic pathways. Conversely, NP-Dox and NP-Dox@M1 exhibited an apparent decrease in the level of pro-caspase-3 and pro-caspase-8, thus indicating the activation of caspase-dependent apoptosis, particularly upon light irradiation.

After light irradiation, NP-Dox@M1 exhibited the most significant downregulation of the antiapoptotic protein BCL-2. Simultaneously, the total levels of the PARP protein were significantly reduced in NP-Dox@M1. BCL-2 and total PARP were down-regulated in light-irradiated groups as compared to the respective nonirradiated groups, further indicating increased apoptotic signaling. A stronger apoptotic effect was noted in the NP-Dox and NP-Dox@M1 groups, as indicated by the substantial down-regulation of the levels of pro-caspase-3 and pro-caspase-8 when compared to the levels of pro-caspase-3 and pro-caspase-8 in the nonirradiated groups. The decrease in the procaspase forms implies increased processing and activation of caspase, which implies that light irradiation treatment additionally induced the caspase cascade and increased the induction of apoptosis in these groups.

These results indicate that light-mediated PDT was the most effective at triggering apoptotic pathways, and the NP-Dox@M1 nanoplatform caused the most significant changes in the expression of the apoptotic proteins, which were the inhibition of BCL-2 and the activation of the caspase-mediated apoptotic cascade.

#### Macrophage Uptake

The clearance behavior of the developed nanoplatforms was tested on the RAW 264.7 macrophage cells, where cellular uptake and intracellular retention of the developed nanoplatforms were examined (Figure 5A,B). The free drug formulations and the respective nanoplatforms were incubated with the cells for 4 h, and the cells were subjected to fluorescence imaging. Fluorescence microscopy was used to analyze intracellular fluorescence signals under the red channel, and quantitative analysis was done using ImageJ.

**Figure 5:**
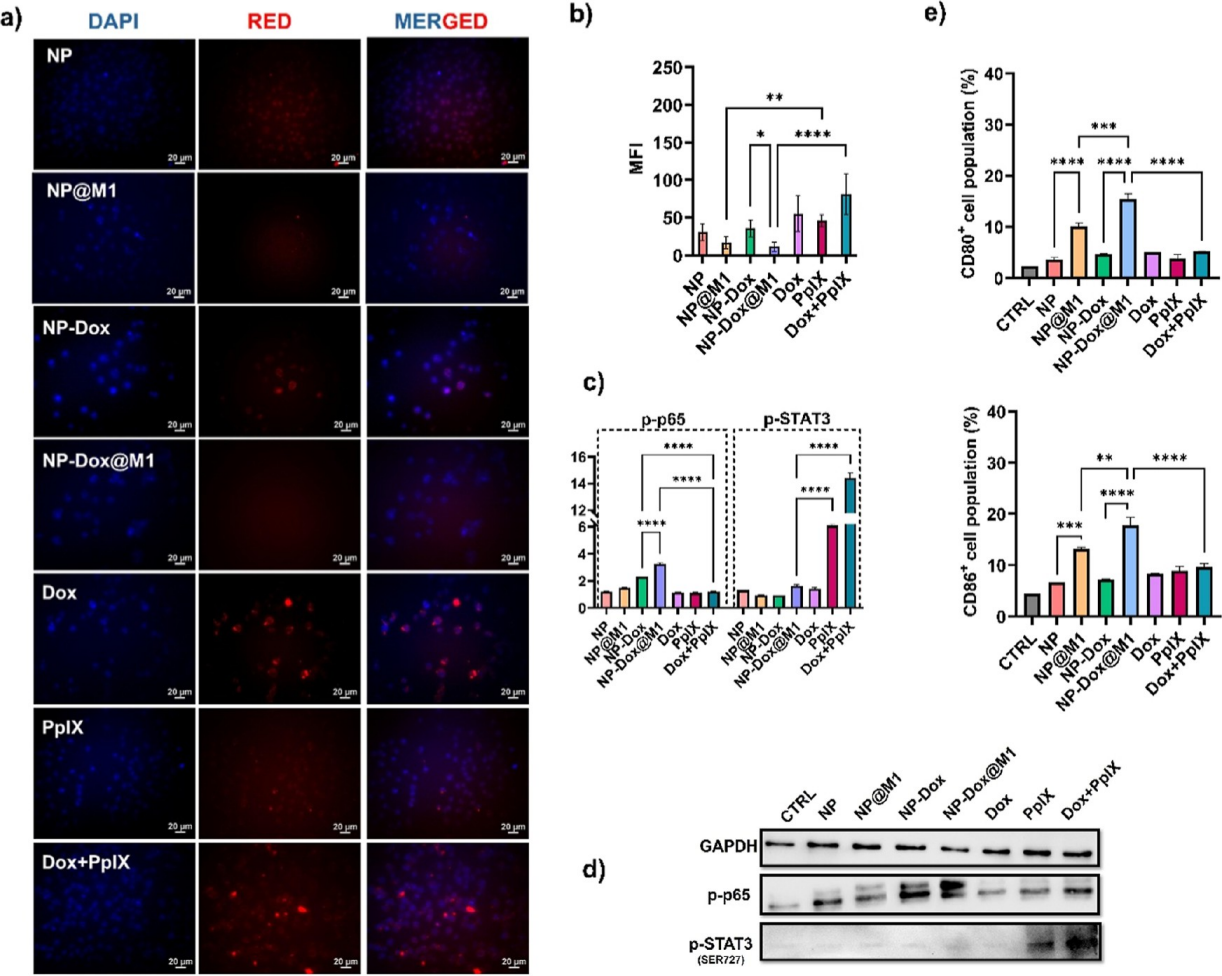
(a) Fluorescence microscopy images after 4 h treatment with NPs and free drugs of RAW264.7 macrophage cells. (b) MFI values obtained from fluorescence microscopy analysis of NPs- and free drugs-treated RAW264.7 cells. (c) Densitometric quantification of complementary activation related proteins normalized to GAPDH and expressed relative to CTRL. (d) Representative immunoblots of indicated proteins with GAPDH as a loading control, (e) CD80 and CD86 positive macrophage cell population according to flow cytometry analysis. Data are presented as means ± SD; *p ≤ 0.05, **p ≤ 0.01, ***p ≤ 0.001, and ****p ≤ 0.0001.

The analysis using fluorescence microscopy showed that the Dox + PpIX group recorded the highest intracellular red fluorescence levels in all of the treatment groups, which means that the freely administered agents were accumulated by the cells faster than the nanoparticle-based formulations. The macrophage-membrane-coated preparation had a comparatively lower intracellular signal of fluorescence in relation to the uncoated nanoparticles and free drugs. This observation indicates that membrane camouflage can minimize macrophage internalization, possibly through self-recognition sites that dampen phagocytic uptake. In general, the lower internalization of the membrane-coated nanoplatform facilitates its capability of avoiding early macrophage elimination, which is favorable for long circulation and enhanced delivery efficiency.

#### Complementary Activation-Related Immunoblotting Assay

Increased phosphorylation of NF-κB p65 and STAT3 (S727) in macrophages can be considered a significant indicator of cellular responses occurring under inflammatory microenvironmental conditions where the complement system is activated. Phosphorylation of NF-κB p65 in macrophages generally indicates activation of the NF-κB pathway and the cell’s transition to a pro-inflammatory transcriptional program.^56^ The increase in p-p65 protein levels suggests that an inflammatory response is triggered in the macrophage, and this is an activation signal consistent with M1-like polarization.^57^ Immunoblotting assay results show that NP-Dox and NP-Dox@M1 cause a significant increase in p-p65 levels (Figure 5C,D).

Phosphorylation of STAT3 at Ser727 (S727) in macrophages is associated with STAT3 gaining full transcriptional activity and more strongly regulating the gene expression. This phosphorylation indicates increased STAT3 activation, and increased STAT3 activation is often associated with anti-inflammatory/immunosuppressive responses.^58^ Frequent M2-like polarization in macrophages can be part of signals supporting immune tolerance and a tumor-promoting phenotype. A significant increase in p-STAT3 levels was observed in the PpIX and Dox + PpIX groups. In contrast, NPs did not significantly increase p-STAT3 levels (Figure 5C,D). All of these results indicate that NPs increase the inflammatory response and promote M1-type activation, while free drug formulations cause an immunosuppressive response.

#### Macrophage Polarization

RAW264.7 cells were incubated with the respective nanoplatforms and free drugs in a test duration of 4 h to assess the influence of the nanoplatforms and free drugs on macrophage polarization (Figures S12, 5E). After incubation, the cells were stained using the CD80-FITC and CD86-PE antibodies as surface markers of M1 macrophages and analyzed using the flow cytometer.

Flow cytometric analysis showed that the treatment of NP@M1 and NP-Dox@M1 caused changes in the expression of M1-related surface markers. Specifically, the tendency of polarization toward M1 was determined by the percentage of CD80^+^ and CD86^+^ cell populations. These findings show that the biomimetic nanoplatforms covered with membranes regulated the macrophage phenotype, implying its possible effects on macrophage activation and polarization to an M1-like macrophage.

## Conclusion

In this study, we successfully developed a cholesterol-assisted, membrane-engineered glycopolymer-based biomimetic nanoplatform coloaded with Dox and PpIX for synergistic treatment of triple-negative breast cancer. Macrophage cells were differentiated to the M1 phenotype to retain potential immunomodulatory advantages. Incorporation of cholesterol into the polymeric core enabled stable hydrophobic insertion and the effective integration of the M1 macrophage membrane, resulting in a biomimetic formulation with enhanced structural stability and functional membrane camouflage. Furthermore, the sugar-based polymer has offered an advanced and effective treatment option by targeting the increased GLUT-5 receptor in TNBC cells.

Comprehensive in vitro evaluations demonstrated that the M1 membrane-engineered nanoplatform exhibited superior cellular uptake compared to the uncoated formulation, and free drug combination also significantly enhanced cytotoxicity against TNBC cells, particularly under light irradiation. Mechanistic investigations confirmed that the therapeutic efficacy was associated with caspase-mediated apoptosis. Moreover, macrophage polarization analysis indicated that the nanoplatform promoted an M1-like phenotype, which highlights its potential therapeutic advantages. Also, it reduced clearance-related internalization in macrophage cells, supporting the immune-evasive characteristics of the membrane-engineered system.

These findings demonstrate that the membrane-engineered multifunctional biomimetic nanoplatform with enhanced chemo-photodynamic synergism has improved intracellular delivery, advanced therapeutic efficacy, and immune-related benefits. This platform holds significant potential for future development as an effective therapeutic approach to triple-negative breast cancer.

## Supplementary Material



## Notes

The characterizations of the monomers; precursor polymers; and raw files of in vitro cell studies (PDF)
