# Supplementary material for: Macrophage Membrane-Engineered Biomimetic Nanoplatform Enables Immune Evasion and Immunomodulation for Enhanced Chemo-Photodynamic Therapy
Source: Biomacromolecules. 2026 Jun 26;27(7):4909–25. doi: 10.1021/acs.biomac.6c01045 (PMC13370769; doi:10.1021/acs.biomac.6c01045)
Supplement: Supplementary file 1 [file bm6c01045_si_001.pdf]

SUPPORTING INFORMATION

Macrophage Membrane-Engineered Biomimetic Nanoplatform Enables Immune Evasion and Immunomodulation for Enhanced Chemo-Photodynamic Therapy

Tugba Gencoglu-Katmerlikaya,[a] Aydan Dag,[b,c*]

[a]Department of Biotechnology, Institute of Health Sciences, Bezmialem Vakif University, 34093, Istanbul, Turkey
[b]Department of Pharmaceutical Chemistry, Faculty of Pharmacy, Bezmialem Vakif University, 34093, Istanbul, Turkey
[c]Pharmaceutical Application and Research Center, Bezmialem Vakif University, 34093, Istanbul, Turkey,

E-mail: adag@bezmialem.edu.tr

Contents

1. Materials and methods

2. Characterization

Gel Permeation Chromatography (GPC)

Nuclear Magnetic Resonance (NMR) Spectroscopy

UV-Vis Spectroscopy

Fourier-Transform Infrared (FT-IR) Spectroscopy

Dynamic Light Scattering (DLS)

Transmission Electron Microscopy (TEM)

3. Synthetic Procedures

Synthesis of Precursor Molecules

4. Supplementary Figures

Supplementary Fig.1: a) ^{1}H NMR and **b)** ^{13}C NMR of cholesterol methacrylate (CMA) monomer.

Supplementary Fig.2: a) ^{1}H NMR and **b)** ^{13}C NMR spectra of *tert*-butoxy carbonyl-protected hydrazine methacrylate (*b*HMA) monomer.

Supplementary Fig.3: a-b) The ^{1}H NMR spectra ($CDCl_3$), **c-d)** GPC traces and **e)** FT-IR spectra of P(*i*FMA) (P1) and P(*i*FMA-*b*-(CMA-*co*-*b*HMA)) (P2).

Supplementary Fig.4: a) The ^{1}H NMR spectra (DMF-d7) and GPC traces of P(*i*FMA-*b*-(CMA-*co*-*b*HMA))-PpIX (P3), **b)** P(FMA-*b*-(CMA-*co*-HMA))-PpIX (P4) and **c)** P(FMA-*b*-(CMA-*co*-HMA-Dox))-PpIX (P5)

Supplementary Fig.5: a) The FT-IR spectra of P(*i*FMA-*b*-(CMA-*co*-*b*HMA))-PpIX (P3), P(FMA-b-(CMA-co-HMA))-PpIX (P4) and P(FMA-*b*-(CMA-*co*-HMA-Dox))-PpIX (P5), **b)** the UV spectra of P(FMA-*b*-(CMA-*co*-HMA))-PpIX (P4) and P(FMA-*b*-(CMA-*co*-HMA-Dox))-PpIX (P5), and **c)** UV calibration curves of PpIX and Dox.

Supplementary Fig.6: Time-dependent DPBF quenching curves indicating ROS generation by P4 and PpIX in DMF under 635 nm light irradiation (100 mW/cm²).

Supplementary Fig.7: a) Flow cytometric analysis of untreated and **b)** 0.5 ug/mL LPS and IFNγ treated RAW264.7 macrophage cells stained with FITC conjugated CD80 and PE conjugated CD86 antibodies.

Supplementary Fig.8: a) Relative cell viability percentages of 3T3-L1 and **b)** 4T1 cells treated with different concentrations of NPs and free drugs for 24 h with (+) or without (-) light exposure determined by MTT assay.

Supplementary Fig.9: Fluorescence microscopy images of NP-Dox@M1, NP-Dox and Dox+PpIX treated 4T1 cells after 2 and 4 h preincubated with or without free Fructose (Fru+ and Fru-).

Supplementary Fig.10: Flow cytometric analysis of 4T1 cells treated with NPs and free drugs for 24 h with or without light exposure stained with PI in the presence of RNAse A.

Supplementary Fig.11: Flow cytometric analysis of 4T1 cells treated with NPs and free drugs for 24 h with or without light exposure stained with FITC conjugated Annexin V and 7-AAD.

Supplementary Fig.12: Flow cytometric analysis of RAW264.7 macrophage cells treated with NPs and free drugs for 24 h stained with FITC conjugated CD80 and PE conjugated CD86 antibodies.

1. Materials and Methods

Protoporphyrin IX (PpIX), 1-Ethyl-3-(3-dimethylaminopropyl)-carbodiimide hydrochloride (EDC.HCl, ≥98.0%), 1-hydroxybenzotriazole hydrate (HOBT, ≥97.0%), N,N, diisopropylethylamine (DIEA), 4',4-azobis (4-cyanopentanoic acid) (ACVA, ≥98.0%), 4-(dimethylamino)pyridine (DMAP, ≥99%), sodium hydroxide pellet (ACS Reagent, ≥97%), borontrifluoride diethyl etherate (for synthesis), D-(-)-fructose (≥99.0%), acetic anhydride (Reagentplus® ≥99.0%), 2,2'-azobis(2-methylpropionitrile) (AIBN, 98.0%), copper bromide (CuBr2, 98%), sodium azide (NaN_3, ≥99.5%), 2-bromoethanol (95%), potasium ferricyanide (III) ($K_3Fe(CN)_6$), elemental sulfur, benzyl chloride (99%), methacryloyl chloride (97%), p-toluensulfonyl chloride (≥99.0%), *N,N,N',N'',N''*-pentamethyldiethylenetriamine (PMDETA,99%), triethylamine (Et_3N, ≥99.0%), N,N-dimethyl formamide (DMF, ≥%99,8), petroleum spirit (ACS Reagent ≥90.0%), dichloromethane (DCM, for HPLC ≥99.8%), tetrahydrofurane (THF, ≥99.9%), diethyl ether (≥99.5%), 2,2,2-Trifluoroethanol (TFE, ReagentPlus®, ≥99%) and chloroform (≥99.4%) were purchased from Sigma Aldrich. Methanol (MeOH, ≥99.5%), acetonitrile (for liquid chromatography≥99.9%), pyridine (EMSURE ACS Reagent ≥99.5%), toluene (EMSURE, for analysis), hydrochloric acid (HCl,

EMPLURA ≥32.0%) and 4 Å molecular sieve were purchased from Merck. Ethyl acetate (EtOAc, ≥99.7% for LC-MS), n-hexane (≥96.0%), sodium bicarbonate (NaHCO3, ≥99.0%), sodium sulfate (Na_2SO_4), sodium chloride (NaCl) were purchased from ISOLAB. 3-(Trimethylsilyl) 1-propanol (97%) was purchased from TCI. Sodium acetate (ACS Reagent ≥99.0%) was purchased from Alfa Aesar. 3-(4,5-Dimethyltiazole-2-yl)-2,5-difenyltetrazolium bromide (MTT), phosphate bovine serum tampon solution (PBS, tablet for 100 mL) was purchased from Invitrogen. 3.5 kDa dialysis membrane was purchased from Repligen. FITC conjugated CD80, PE conjugated CD86 antibodies and FITC-Annexin V /7-AAD apoptosis kit were purchased from Biolegend. All remaining chemicals were purchased from Sigma Aldrich and used without further purification.

2. Characterization

Gel Permeation Chromatography (GPC)

The molecular weight and polydispersity indices of synthesized polymers were analyzed by gel permeation chromatography (GPC). DMF-GPC analyses were performed on a TOSOH EcoSEC/GPC system equipped with a refractive index (RI) detector, a UV detector, a DLS Wyatt Dynapro Nanostar detector, and a DAWN HELEOS-18 multi-angle laser light scattering detector, (MALLS detector, Wyatt Technology, Santa Barbara, CA, USA). DMF (0.01 M LiBr, HPLC grade) with a flow rate of 0.5 mL/min at 45 °C was used as the mobile phase. DMF-GPC analyses were performed using serially connected size-exclusion columns (TSKgel HHR guard column, G3000 HHR, and G5000 HHR (7.8 mm ID x 30 cm)). PMMA standards were used for calibration, and molecular weight and polydispersity indices were determined using either OPUS or TOSOH GPC software.

Nuclear Magnetic Resonance (NMR) Spectroscopy

NMR analyses were conducted using a Bruker BioSpin AG Avance 500 MHz Spectrometer (^{1}H (500 MHz), ^{13}C (125 MHz)). All chemical shifts are expressed in parts per million (ppm) (δ) relative to $Si(CH_3)_4$ as the internal standard (δ = 0 ppm), referenced to the chemical shifts of characteristic solvent signals (^{1}H and ^{13}C).

UV-Vis Spectroscopy

UV-Vis spectra were recorded on a Hitachi U-2900 UV-Vis spectrophotometer.

Fourier-Transform Infrared (FT-IR) Spectroscopy

FT-IR analyses of samples were performed on a Bruker Alpha infrared spectrometer equipped with an attenuated total reflectance (ATR) device and germanium crystal. FT-IR spectra of polymers were recorded within a wave number range of 400-4000 cm^{-1} with a resolution of 4 cm^{-1} from 100 scans in transmission mode. The spectra were determined using both the “OPUS” and Graphpad Prism v10 software.

Dynamic Light Scattering (DLS)

Nanoparticle sizes (the average hydrodynamic diameters and size distributions of the prepared nanoparticle solutions) were determined using a Malvern Zetasizer Nano ZS particle size analyzer equipped with a 4 mW He-Ne laser operating at λ = 632 nm, an avalanche photodiode detector with high quantum efficiency, and an ALV/LSE-5003 multiple tau digital correlator

electronics system. Samples were prepared at a concentration of 1 mg/mL in UP water and purified from dust using a microfilter (0.45 μm) before the measurements.

Transmission Electron Microscopy (TEM)

TEM micrographs were obtained using a FEI TALOS F200S transmission electron microscope. The instrument operates at an accelerating voltage of 200.0 kV. Samples were imaged without any staining. The particles were cast onto a carbon-coated copper grid by placing a droplet, a micelle aqueous solution for 15 min onto its surface. Subsequently, the excess of the solution was removed using filter paper. The grids were dried on air.

Flow Cytometer

Flow cytometric analyses were performed using a Novocyte™ Spectral Flow Cytometer Systems (Agilent Technologies) equipped with 3 lasers, enabling multiparametric detection of fluorescent markers. Data acquisition was carried out using NovoExpress software (Agilent Technologies), and the results were further analyzed using FlowJo software. Each experiment was repeated at least three times.

Fluorescence Microscopy

Fluorescence imaging was performed using a Zeiss Axio Observer Z1 widefield microscope (Carl Zeiss Microscopy) equipped with a Plan-Apochromat objective lens, an LED fluorescence light source (Colibri illumination system), and an AxioCam digital camera (Carl Zeiss). Data acquisition was carried out using Zeiss ZEN 2 imaging software and the results were further analyzed using ImageJ software.

3. Synthetic Procedures

Synthesis of Precursor Molecules

Alkyne functional CPADB RAFT agent[1], isopropylidene protected fructose methacrylate (*i*FMA)[2] monomer and azide functionalized protoporphyrin IX (PpIX-N_3)[3] were synthesized as our previously published procedures. Cholesterol methacrylate (CMA)[4] and tert butoxy carbonyl protected hydrazine methacrylate (*b*HMA)[5] monomers were synthesized according to the procedure existing in the literature.

4. Supplementary Figures

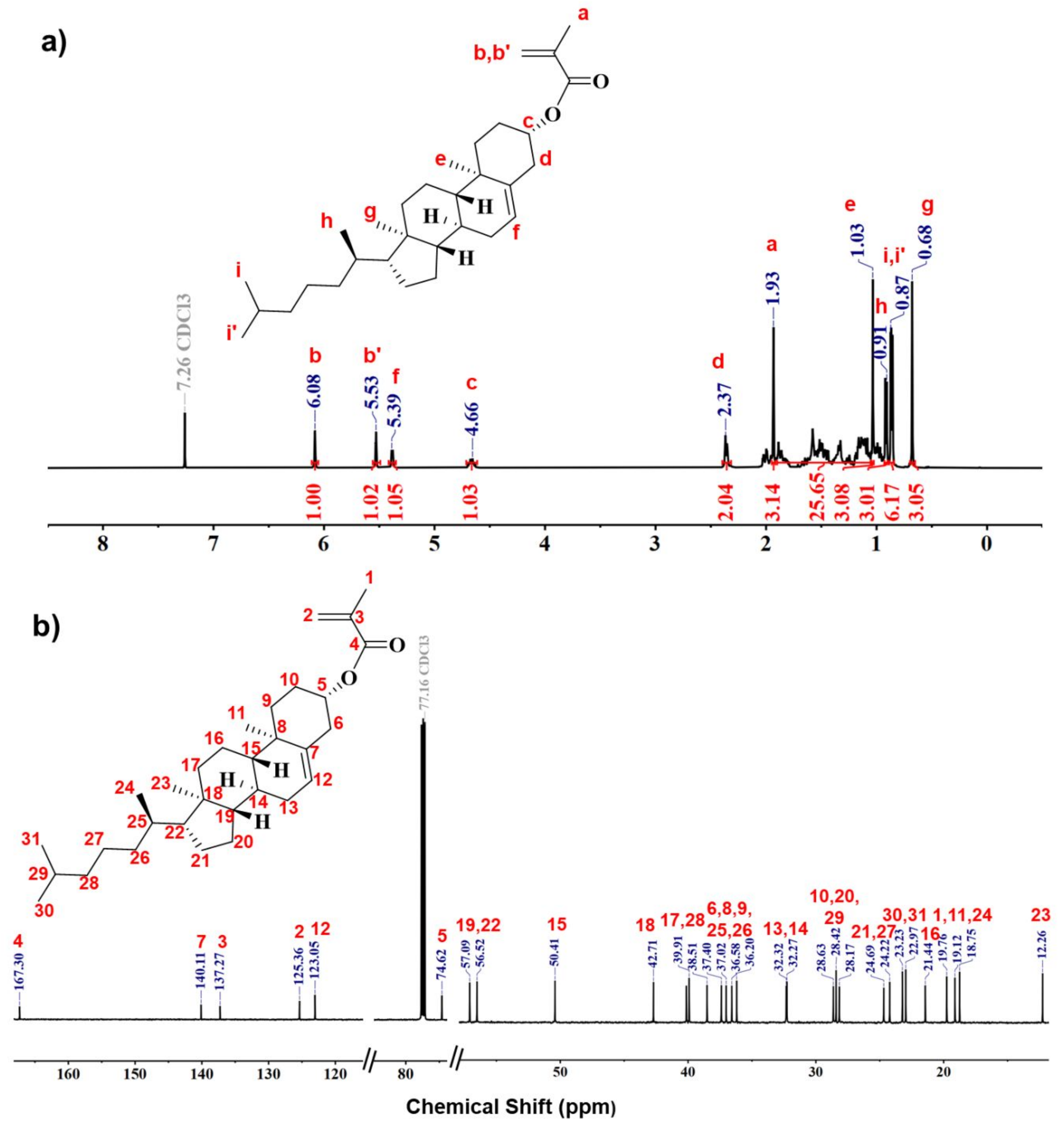

Figure S1: a) ^{1}H NMR and **b)** ^{13}C NMR of cholesterol methacrylate (CMA) monomer.

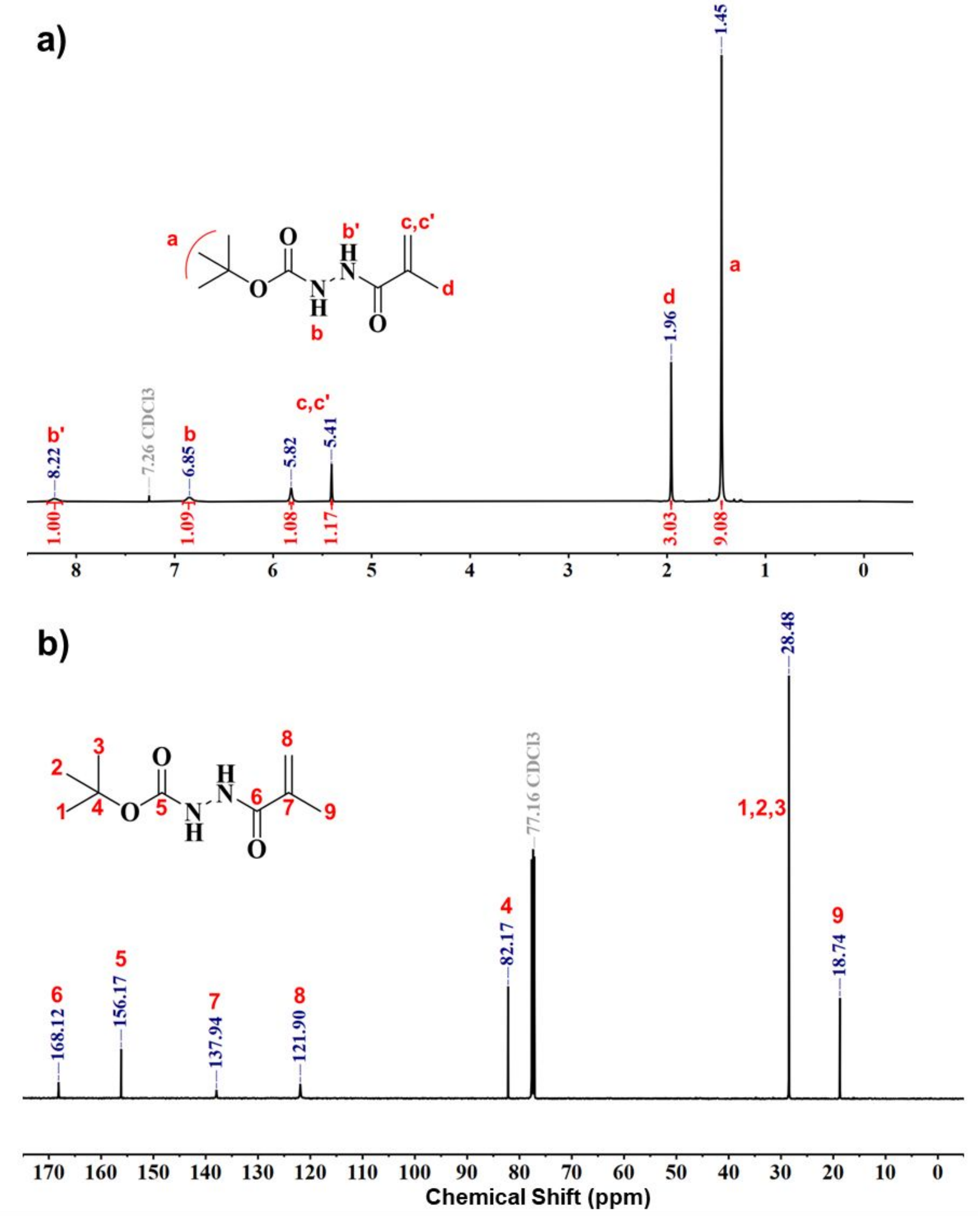

Figure S2: a) ^{1}H NMR and **b)** ^{13}C NMR spectra of *tert*-butoxy carbonyl-protected hydrazine methacrylate (*b*HMA) monomer.

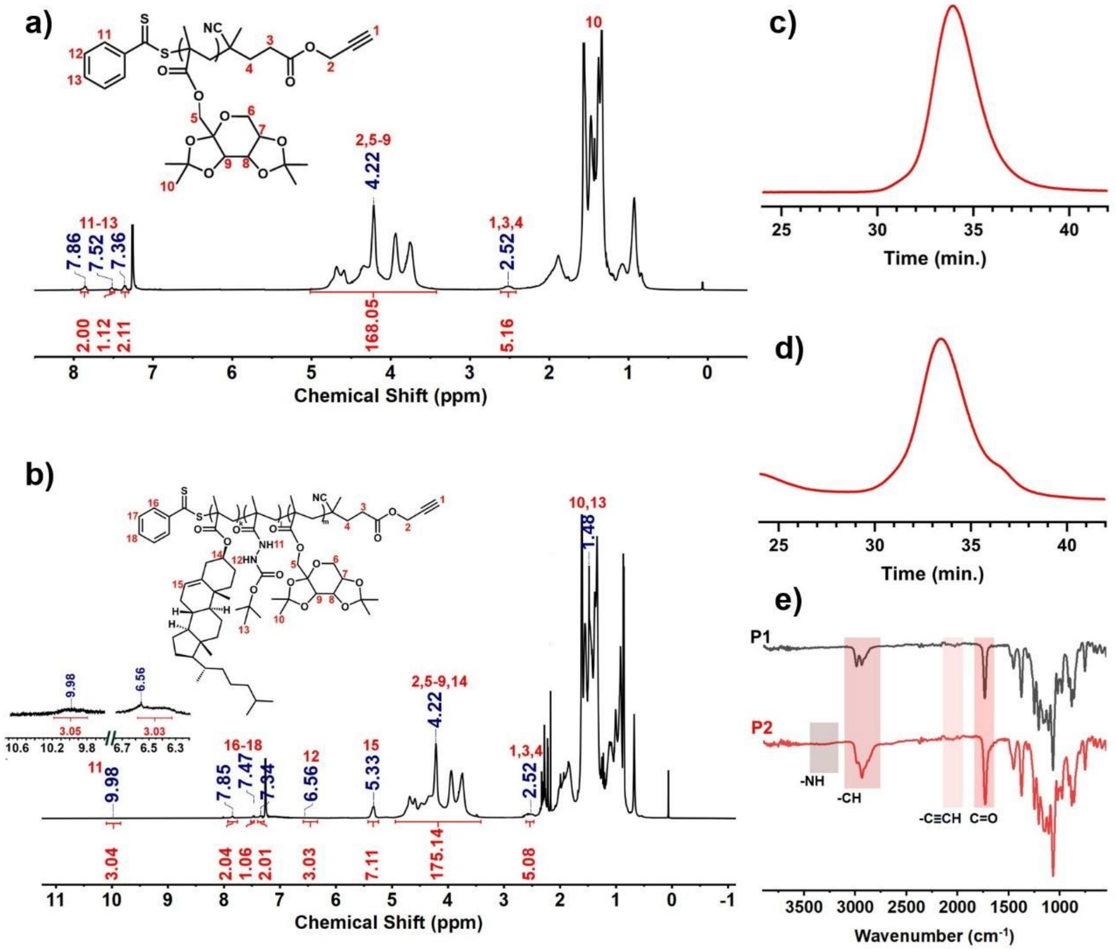

Figure S3: a-b) The ^{1}H NMR spectra ($CDCl_3$), **c-d)** GPC traces, and **e)** FT-IR spectra of P(*i*FMA) (P1) and P(*i*FMA-*b*-(CMA-*co*-*b*HMA)) (P2).

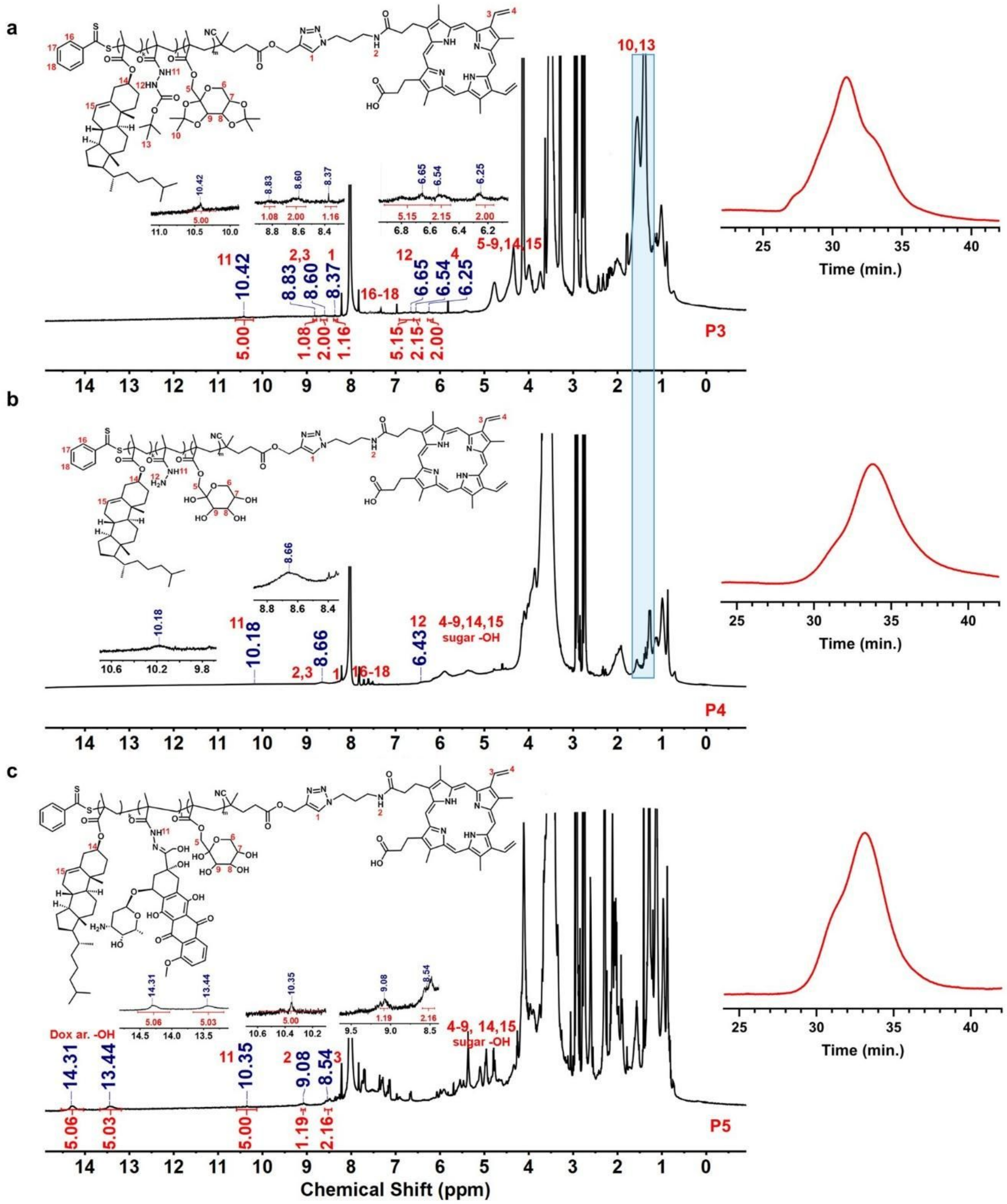

Figure S4: a) The ^{1}H NMR spectra (DMF-d_7) and GPC traces of P(*i*FMA-*b*-(CMA-*co*-*b*HMA))-PpIX (P3), **b)** P(FMA-*b*-(CMA-*co*-HMA))-PpIX (P4) and **c)** P(FMA-*b*-(CMA-*co*-HMA-Dox))-PpIX (P5)

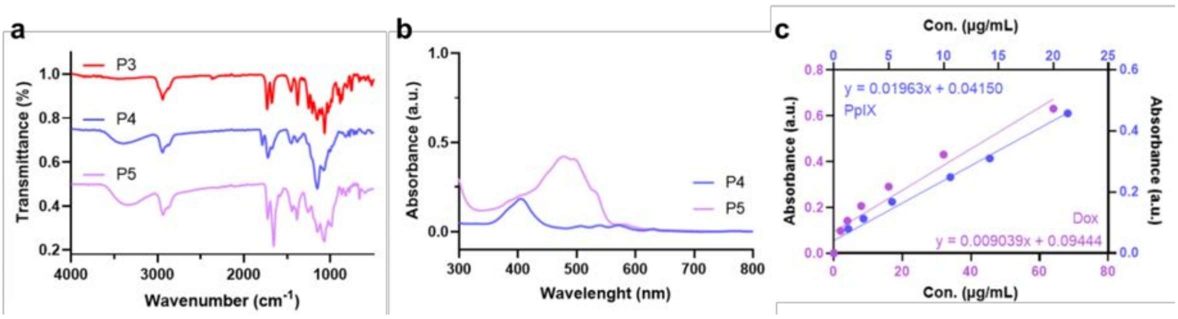

Figure S5: a) The FT-IR spectra of P(*i*FMA-*b*-(CMA-*co*-*b*HMA))-PpIX (P3), P(FMA-*b*-(CMA-*co*-HMA))-PpIX (P4) and P(FMA-*b*-(CMA-*co*-HMA-Dox))-PpIX (P5), **b)** the UV spectra of P(FMA-*b*-(CMA-*co*-HMA))-PpIX (P4) and P(FMA-*b*-(CMA-*co*-HMA-Dox))-PpIX (P5), and **c)** UV-Vis calibration curves of PpIX and Dox.

Supplementary Equation for Singlet Oxygen Quantum Yields:

The singlet oxygen quantum yield of PpIX conjugated polymer (P4) was determined by using the equation given below [6] (1);

$$\Phi\Delta,x = \Phi\Delta,ref \times \left(\frac{k_x}{k_{ref}}\right) \times \left(\frac{A_{ref}}{A_x}\right) \qquad (1)$$

The singlet oxygen quantum yields of the samples ($\Phi_{\Delta,x}$) were determined relative to the reference photosensitizer PpIX ($\Phi_{\Delta,ref}$= 0.73).[7] Here, k_x and k_{ref} represent the 1,3-diphenylisobenzofuran (DPBF) photodegradation rate constants of the sample (P4) and the reference (PpIX), respectively, while Ax and Aref denote their absorbance values. For singlet oxygen generation measurements, DPBF (150 µM) was added to equal-concentration solutions of P4 and PpIX in DMF. The mixtures were irradiated with 635 nm light (100 mW/cm²) for 10 min, and DPBF degradation was monitored by measuring the decrease in its absorbance at 414 nm. The rate constants were obtained from the slopes of the plots of A_t/A_0 versus irradiation time (t), where At is the absorbance of DPBF at a given irradiation time and A_0 is its initial absorbance. The singlet oxygen quantum yields were subsequently calculated from the DPBF degradation rates.

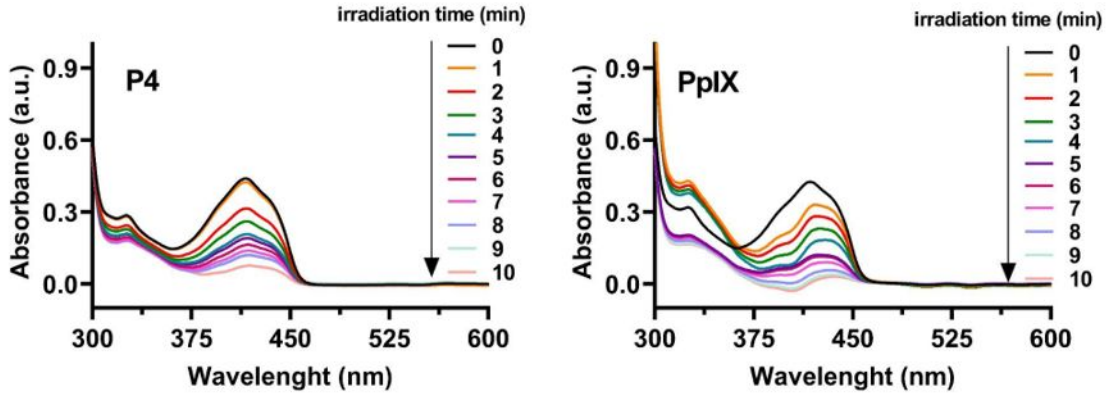

Figure S6: Time-dependent DPBF quenching curves indicating ROS generation by P4 and PpIX in DMF under 635 nm light irradiation (100 mW/cm²**).**

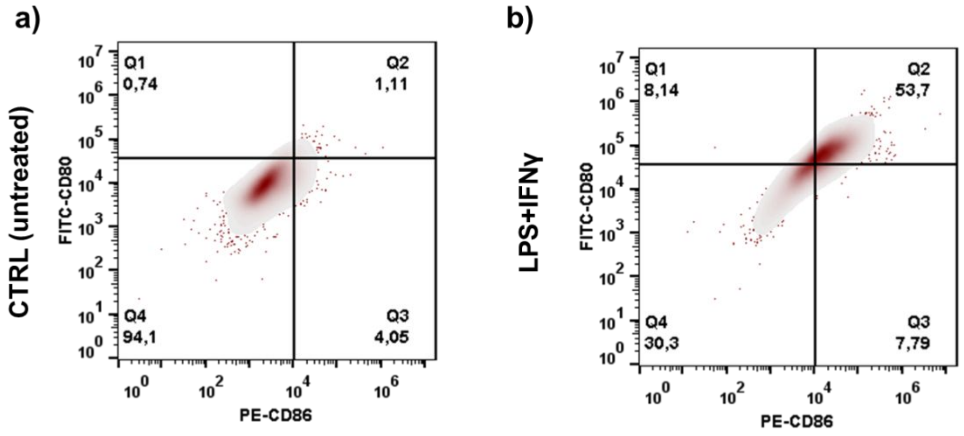

Figure S7: a) Flow cytometric analysis of untreated and **b)** 0.5 µg/mL LPS and IFNγ treated RAW264.7 macrophage cells stained with FITC conjugated CD80 and PE conjugated CD86 antibodies.

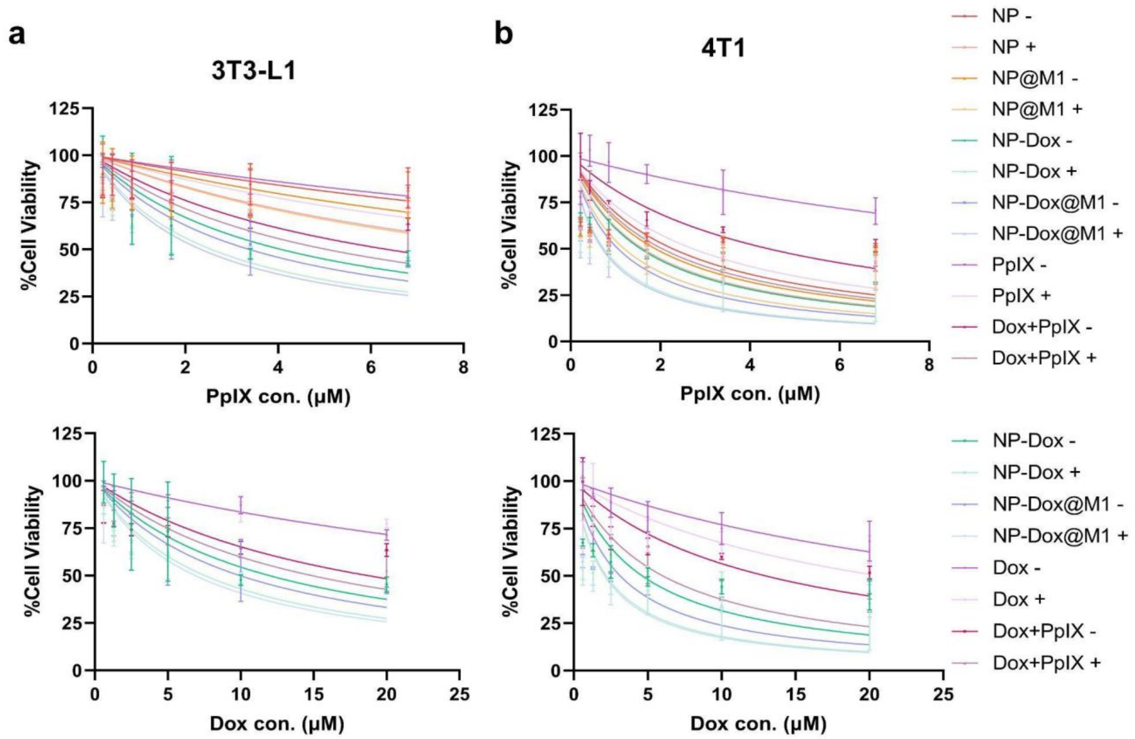

Figure S8: a) Relative cell viability percentages of 3T3-L1 and **b)** 4T1 cells treated with different concentrations of NPs and free drugs for 24 h with (+) or without (-) light exposure determined by MTT assay.

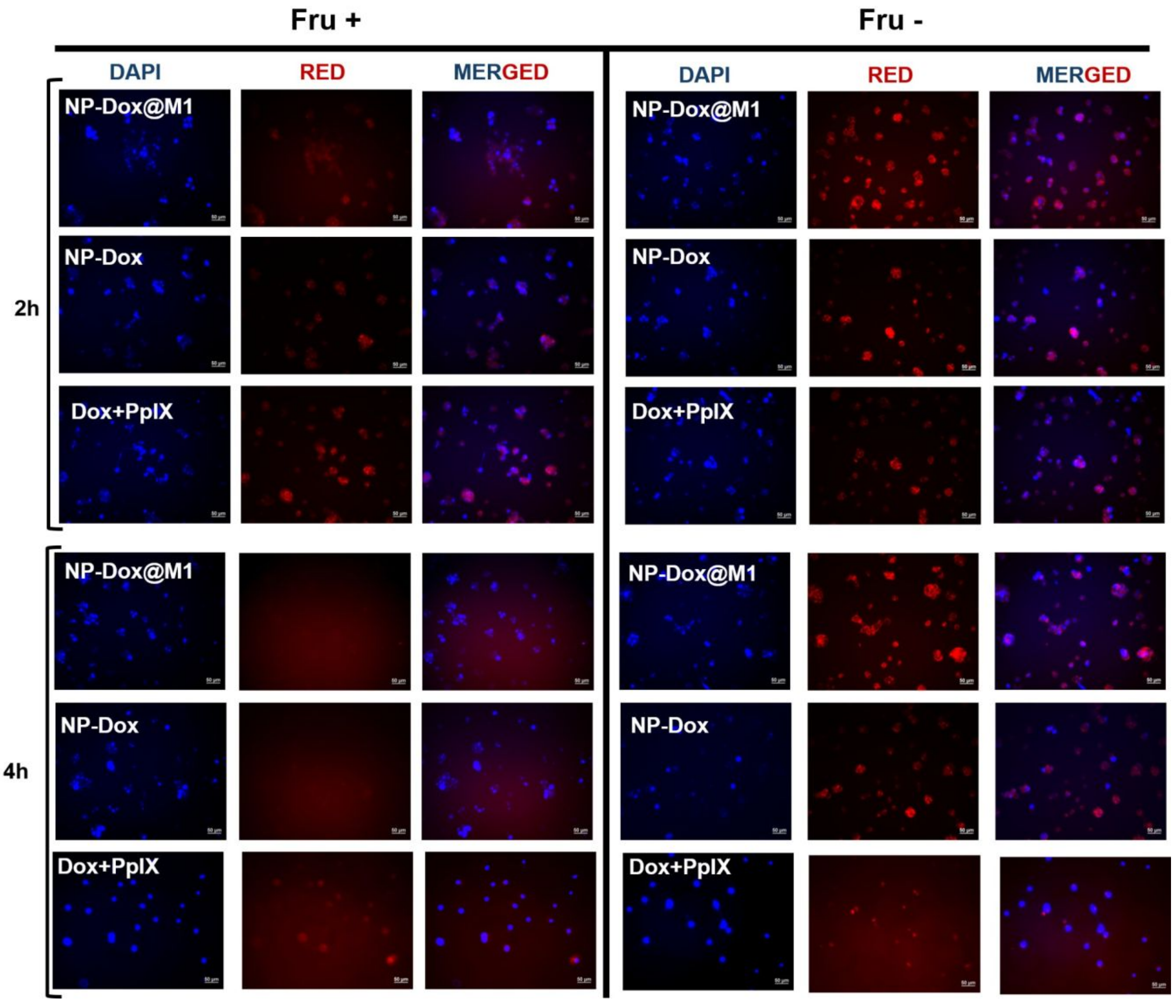

Figure S9: Fluorescence microscopy images of NP-Dox@M1, NP-Dox, and Dox+PpIX treated 4T1 cells after 2 and 4 h preincubated with or without free Fructose (Fru+ and Fru-).

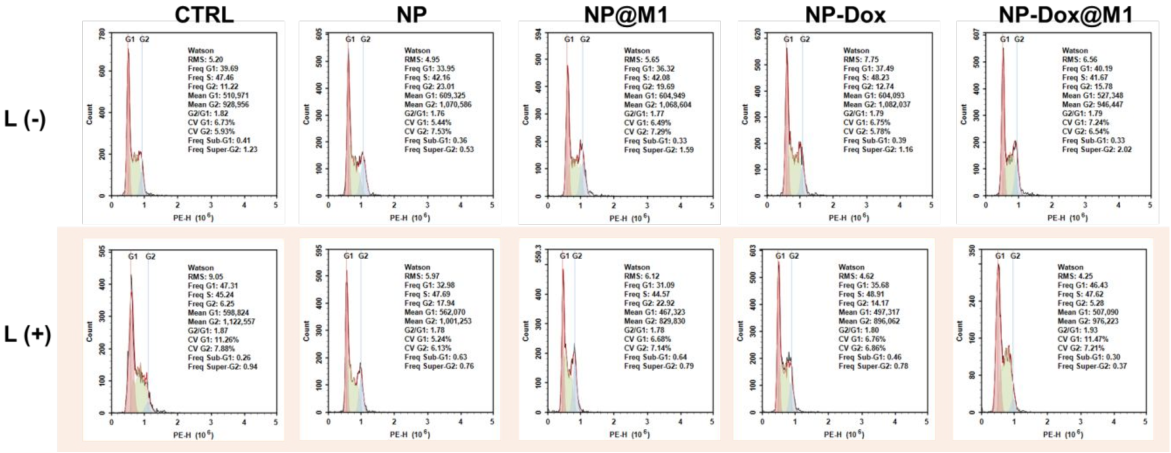

Figure S10: Flow cytometric analysis of 4T1 cells treated with NPs and free drugs for 24 h with or without light exposure stained with PI in the presence of RNAse A.

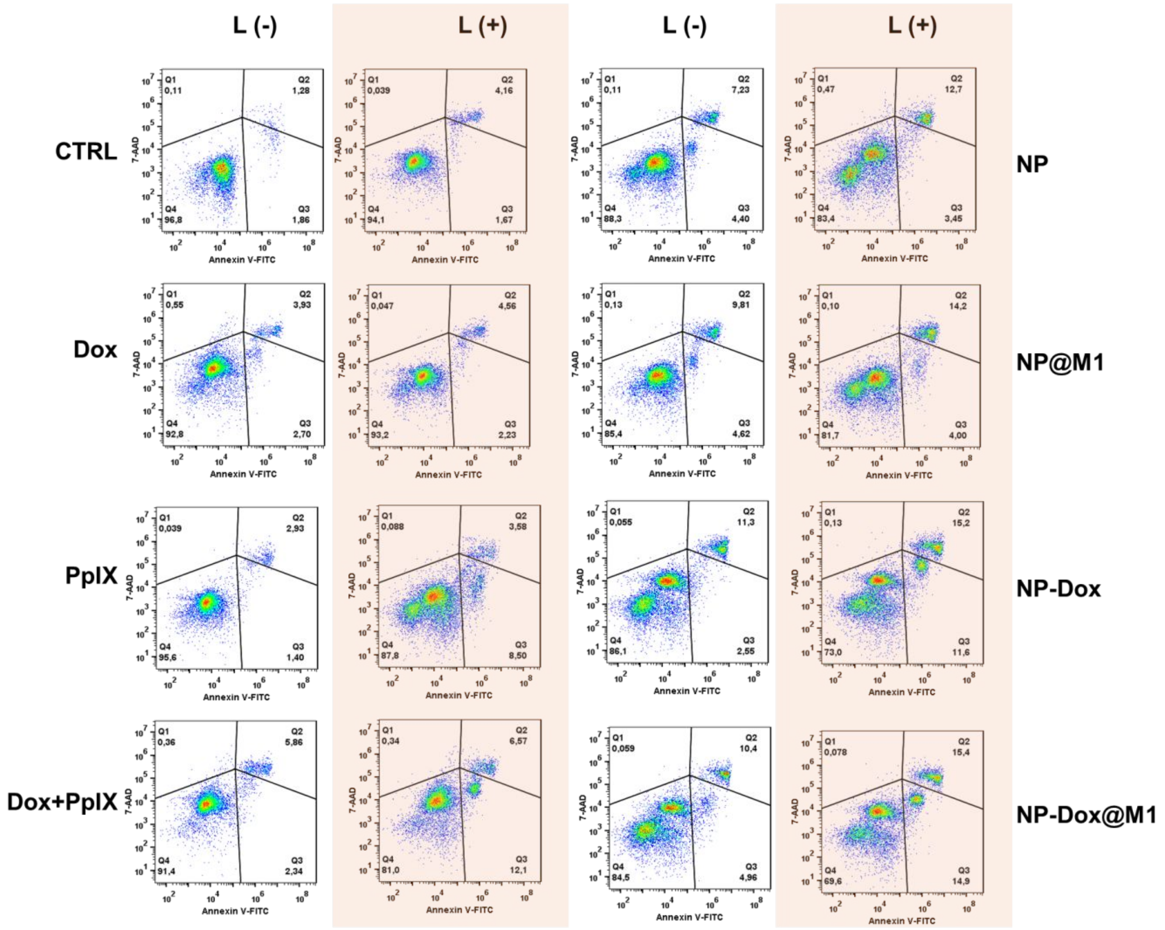

Figure S11: Flow cytometric analysis of 4T1 cells treated with NPs and free drugs for 24 h with or without light exposure stained with FITC conjugated Annexin V and 7-AAD.

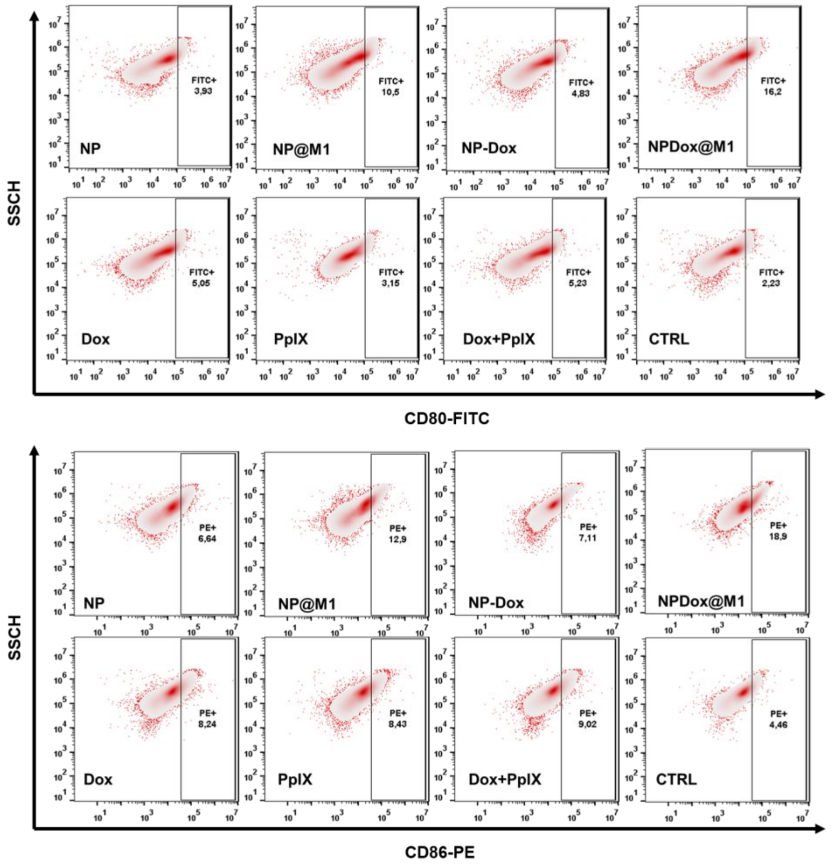

Figure S12: Flow cytometric analysis of RAW264.7 macrophage cells treated with NPs and free drugs for 24 h stained with FITC conjugated CD80 and PE conjugated CD86 antibodies.

References

1. Atasoy, S., Katmerlikaya, T. G., Sancakli, B., & Dag, A. (2025). Remote-controlled release of therapeutics from multifunctional glycoplexes inhibit melanoma cells. European Polymer Journal, 226, 113726.

2. Katmerlikaya, T. G., Dag, A., Ozgen, P. S. O., & Ersen, B. C. (2022). Dual-drug conjugated glyco-nanoassemblies for tumor-triggered targeting and synergistic cancer therapy. ACS Applied Bio Materials, 5(11), 5356-5364.

3. Gencoglu Katmerlikaya, T., Cetin Ersen, B., Dag, A., Sancakli, B., Omurtag Ozgen, P. S., Keskin Yalcin, E., & Avci, B. (2024). Glycopolymeric Photoactive Micelles for Glucose Transporter-Targeted Synergistic Combination Therapy. ACS Applied Polymer Materials, 6(7), 4149-4163.

4. Sevimli, S., Inci, F., Zareie, H. M., & Bulmus, V. (2012). Well-defined cholesterol polymers with pH-controlled membrane switching activity. Biomacromolecules, 13(10), 3064-3075.

5. Zhao, Y., Houston, Z. H., Simpson, J. D., Chen, L., Fletcher, N. L., Fuchs, A. V., ... & Thurecht, K. J. (2017). Using peptide aptamer targeted polymers as a model nanomedicine for investigating drug distribution in cancer nanotheranostics. Molecular pharmaceutics, 14(10), 3539-3549.

6. Kou, M., Qin, F., Zhang, Z., & Li, L. (2025). Methodology for determining singlet oxygen quantum yield in dimethyl sulfoxide. *Analytica Chimica Acta*, 344886.

7. Kozlikova, M., Alfred, M. A., Machacek, M., Dumoulin, F., de la Escosura, A., Goslinski, T., ... & Novakova, V. (2026). Identifying Structural Factors Governing the Photodynamic Activity of Phthalocyanines. *Journal of Medicinal Chemistry*.
